# Systems-level decoding reveals the cognitive and behavioral profile of the human intraparietal sulcus

**DOI:** 10.3389/fnimg.2022.1074674

**Published:** 2023-01-09

**Authors:** Ole Jonas Boeken, Sebastian Markett

**Affiliations:** Department of Molecular Psychology, Institute for Psychology, Humboldt-Universität zu Berlin, Berlin, Germany

**Keywords:** resting state functional connectivity, intraparietal sulcus, systems-level decoding, neuroinformatics, Neurosynth

## Abstract

**Introduction:**

The human intraparietal sulcus (IPS) covers large portions of the posterior cortical surface and has been implicated in a variety of cognitive functions. It is, however, unclear how cognitive functions dissociate between the IPS's heterogeneous subdivisions, particularly in perspective to their connectivity profile.

**Methods:**

We applied a neuroinformatics driven system-level decoding on three cytoarchitectural distinct subdivisions (hIP1, hIP2, hIP3) per hemisphere, with the aim to disentangle the cognitive profile of the IPS in conjunction with functionally connected cortical regions.

**Results:**

The system-level decoding revealed nine functional systems based on meta-analytical associations of IPS subdivisions and their cortical coactivations: Two systems–working memory and numeric cognition–which are centered on all IPS subdivisions, and seven systems–*attention, language, grasping, recognition memory, rotation, detection of motions/shapes* and *navigation*–with varying degrees of dissociation across subdivisions and hemispheres. By probing the spatial overlap between systems-level co-activations of the IPS and seven canonical intrinsic resting state networks, we observed a trend toward more co-activation between hIP1 and the front parietal network, between hIP2 and hIP3 and the dorsal attention network, and between hIP3 and the visual and somatomotor network.

**Discussion:**

Our results confirm previous findings on the IPS's role in cognition but also point to previously unknown differentiation along the IPS, which present viable starting points for future work. We also present the systems-level decoding as promising approach toward functional decoding of the human connectome.

## 1. Introduction

The human intraparietal sulcus separates the superior (SPL) from the inferior parietal lobule (IPL) within posterior parietal cortex (Binkofski et al., 2016). The IPS is relatively large, covering approximately 17% of the parietal lobe's surface, and can be divided into at least three distinct areas with distinct cytoarchitecture and structural and functional connectivity patterns (Scheperjans et al., 2008a; Uddin et al., 2010; Glasser et al., 2016): hIP1 and the hIP2 cover the most anterior aspects of the IPS (Choi et al., 2006) whereas the hIP3 is located posteriorly to hIP1 and hIP2 (Scheperjans et al., 2008a,b). A large body of literature has addressed the IPS's role in a variety of cognitive functions, yet it remains unclear how different cognitive functions map onto the distinct subdivisions within the IPS.

The IPS has been implicated in many cognitive functions. Among these are numerical cognition, including both: the processing of numerical magnitudes and arithmetic operations (Dehaene et al., 2003; Ansari, 2008; Wu et al., 2009; Pinel and Dehaene, 2010; Arsalidou and Taylor, 2011; He et al., 2015; Liu et al., 2017; Vogel et al., 2017; Price et al., 2018; Castaldi et al., 2020; Roell et al., 2021). Activations within IPS were also found during memory routines. These include encoding and maintaining of visual information in visual short-term memory tasks (Xu and Chun, 2006; Harrison et al., 2010; Offen et al., 2010; Domijan, 2011; Xu and Jeong, 2015; Markett et al., 2018; Sheremata et al., 2018; Duma et al., 2019; Praß and de Haan, 2019; Lefco et al., 2020) as well as familiarity based memory retrieval (Frithsen and Miller, 2014; Hutchinson et al., 2014; Rosen et al., 2015, 2018; Chen et al., 2017). Further, the IPS engages in visuomotor coordination such as grasping, action observation and control of hand and eye movements (Corbetta et al., 1998; Shikata et al., 2001; Grefkes and Fink, 2005; Villarreal et al., 2008; Cavina-Pratesi et al., 2018). This engagement extends to visuospatial processes including navigation and the perception of shapes and motions (Salillas et al., 2009; Tark and Curtis, 2013; Binkofski et al., 2016; Meier et al., 2018; Schultz and Bülthoff, 2019; Li and Shigemasu, 2021). Finally, the IPS plays an important role in the interplay of top-down and bottom-up guided pull of attentional processes (Corbetta et al., 1998, 2008; Corbetta and Shulman, 2002; Katsuki and Constantinidis, 2014; Connolly et al., 2016; Markett et al., 2022). Visuomotor coordination and visuospatial attentional processes are closely tied to saccade planning, in which the IPS is also involved (Corbetta et al., 1998; Grosbras et al., 2005; Morris et al., 2007; Baltaretu et al., 2020). But to the best of our knowledge only few attempts have been made to systematically disentangle the role of the cytoarchitecturally distinct IPS regions in cognitive functioning: While visuomotor functioning (Richter et al., 2019), mathematical operations (Wu et al., 2009; Price et al., 2018; Chang et al., 2019) and visuospatial tasks (Gillebert et al., 2013; Papadopoulos et al., 2018) seem to dissociate between the three IPS regions, no study, however, has covered the whole spectrum of the diverse cognitive operations the IPS is involved in.

With the present study, we aim to systematically disentangle the functional profile of the three cytoarchitecturally distinct IPS regions with Bayesian reverse inference decoding of neuroimaging data in Neurosynth (Yarkoni et al., 2011). The Neurosynth database contains data from thousands of neuroimaging studies, including activation coordinates and a large variety of psychological constructs that allow for a more fine-grained decoding, which facilitates the systematic research on structure-to-function relationships of individual brain regions. Given the IPS' embedding into distinct intrinsic connectivity networks and previous suggestions that the connectivity profile of the IPS is also functionally relevant (Corbetta and Shulman, 2002; Fox et al., 2005; Dosenbach et al., 2007; Corbetta et al., 2008; Power et al., 2011; Vossel et al., 2014), we additionally apply a newly developed systems-level decoding, where we simultaneously decode the functional profile of the different IPS regions and functionally connected cortical regions. This novel decoding strategy is based on co-activations and acknowledges that cognitive processes emerge from the interplay of interconnected regions. It thus complements already existing decoding strategies and should be more sensitive in detecting structure-function relationships. Given that hIP1, hIP2, and hIP3 have shown dissociable connectivity patterns (Uddin et al., 2010), we aim with this systems-level decoding to disentangle the functional spectrum of the three IPS seeds and their participation in putative functional neuronal systems.

## 2. Materials and methods

### 2.1. Ethics approval

All analysis used secondary data and no new human participants were generated. Ethics approval was obtained by the authors of the original datasets from their respective ethics review boards.

### 2.2. Definition of the IPS seed regions

**Figure 1:**
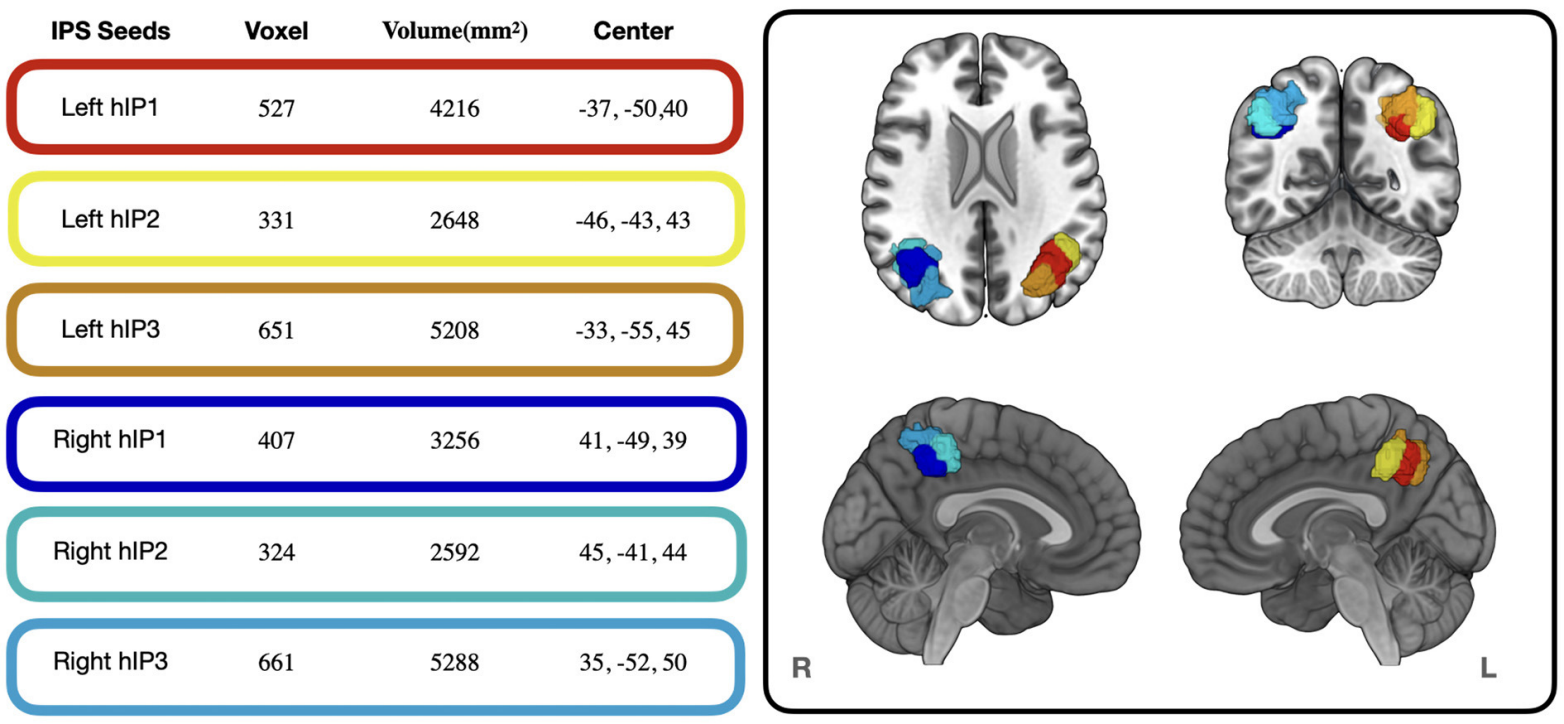
Voxel size, Volume and Center of Mass of the IPS seed regions used in the present study. Colored frames around the values correspond to the colors of the seed regions displayed in the cortical renderings on the right. The cortical renderings were generated with MRIcro GL (Rorden and Brett, 2000).

We used the Anatomy Toolbox (Eickhoff et al., 2005) to define seed regions in the left and right IPS. The IPS parcellation in the Anatomy Toolbox represents probability maps driven by cytoarchitectonic properties (e.g., cell bodies in cortical layers) and subdivides the IPS in three different regions in each hemisphere (Choi et al., 2006; Scheperjans et al., 2008a,b) (see Figure 1). By grounding our seed definition on cytoarchitectonic data, we avoid issues with circularity when analyzing structure-to-function associations (Kriegeskorte et al., 2009). The IPS parcellation within the Anatomy Toolbox are based on an observer-independent mapping approach, which accounts for the well-known interindividual variability of the IPS's sulcal segments (Zlatkina and Petrides, 2014) and facilitates meta-analytic decoding based on neuroimaging data (Richter et al., 2019). Extracted seed regions were “downsampled” to 2 mm with FSL flirt (Jenkinson et al., 2012) and thresholded to include only voxels with probability of >25%.

### 2.3. Resting state data

We used whole-brain seed-based resting-state functional connectivity (RSFC) in combination with a cortical parcellation (Huang et al., 2022) to identify cortical regions with functional coupling to the six IPS seed regions. We utilized resting-state data (~11 min, TR = 800 ms, 2 mm cubic voxels) from a sample of *N* = 84 healthy young adults from a previous study (Markett et al., 2022), acquired with the HCP's pulse sequences (Harms et al., 2018) and preprocessed with HCP's minimal processing pipeline (Glasser et al., 2013) including noise-removal with ICA-Fix. Mean timeseries were extracted from each seed region's set of voxels and regressed against the time courses from all other voxels within the brain mask. The resulting individual-level seed-based RSFC maps were then submitted to a random-effects second level analysis in SPM12 and the group-level maps were thresholded to keep the family-wise error below 5% at the voxel level. From the thresholded maps, we calculated the relative overlap with 360 cortical regions from a volumetric version of HCP's multimodal parcellation and retained lists of functionally coupled cortical regions with at least 80% spatial overlap for each seed regions. Cortical regions overlapping with any seed region were excluded from the lists. Full details on all acquisition and preprocessing steps are described in Markett et al. (2022).

### 2.4. Functional decoding of IPS seed regions

We utilized the NeurosynthDecoder as implemented in the Neuroimaging Meta-Analysis Research Environment [NiMARE; (Salo et al., 2021)] to perform the functional decoding for each of the IPS seed regions. The NeurosynthDecoder allows for reverse inference decoding of activation-term associations, which infers the probability by which different behaviors were executed while a given brain region was active. The (~3,000) terms included in the database are a result from automated parsing through abstracts of published neuroimaging studies (Yarkoni et al., 2011) and concern psychological constructs (e.g., “working memory,” “attention”), anatomical regions (e.g., “intraparietal,” “parietal”) or terms that defy a clear categorization (e.g., “greater extent,” “relied”).

Calculation of reverse inferences provides posterior probabilities of a term given activation of the region *AND* given the prior probability of a term (i.e., the prevalence of the term in the database). Formally, the posterior probability is computed as P(term|activation, p) = p(P(activation|term)/P(activation|term, p) where the prior probability p is set beforehand to 0.5 (i.e., 50% chance of a brain region experiencing the brain state described by the term). This rather conservative approach equates the possibly distinct baserate of terms within the database, which enables the handling of otherwise very different posterior probabilities, that are used for the interpretation of functional decoding results (Yarkoni et al., 2011). For clarification, P(activation|term, p) represents the forward inference and gets computed as P(activation|term, p) = pP(activation|term) + (1p)P(activation|not having the term). In addition to the posterior probabilities (i.e., the effect sizes of the reverse inference decoding), a two-way chi-square was performed to determine if the presence of the label and the selection of a term are statistically independent (<0.05). Correction for multiple comparisons was done by applying a Benjamini-Hochberg FDR correction. More details are given in the NIMARE documentation, accessible at: https://nimare.readthedocs.io/en/latest/decoding.html).

In the present study, we performed the decoding for each individual IPS seed, by submitting each seed separately to the NeurosynthDecoder. This resulted in a list of associated terms and their posterior probabilities. Of these terms, we selected the 30 terms with highest posterior probability for each seed region to ensure comparability. The automatic NeurosynthDecoder lists all terms, irrespective of whether they passed the multiple comparison significance testing or not. We therefore excluded those terms that did not pass the multiple comparison significance testing *post-hoc* using an in-house MATLAB (2021) script.

### 2.5. System-level decoding of IPS seed-cortical pairs

The core operation of the systems decoding includes the simultaneous decoding of a given IPS seed and a functionally connected brain region, from which only those terms are retained that were not already obtained in the functional decoding of the connected region alone. Iterating across all seed-cortical pairs, the NeurosynthDecoder was applied twice: The first step decoded the seed region in conjunction with the cortical region, the second step decoded the cortical region alone. This resulted in two sets of terms which were compared with a *logical-and-conjunction* to produce a list of unique terms present in both sets and a *set difference* to produce a list of terms specific to the IPS seed-cortical pairs. The entire systems-level decoding framework enabled us to generate reverse inference maps for all seed-cortical pairs and to display all brain regions in that seed-cortical system that were associated with a particular term.

#### 2.5.1. Grouping of the systems-level decoding results in Neurosynth topics

The Neurosynth database contains a large variety of terms, including psychological constructs, from which we derived the posterior probability of structure-to-function relationships. To ease interpretability, we aggregated the terms into larger topics, as provided on the Neurosynth webpage (Yarkoni, 2018). In brief, the topics were originally defined by text mining over neuroimaging article's abstracts by using latent Dirichlet allocation (LDA). LDA is a topic modeling technique that is suited to extract hidden (i.e., latent) topics form large corpus of text data (Blei et al., 2003). Details on the topic modeling in Neurosynth are described in the original publication (Poldrack et al., 2012). We used the most recent version of 50 topics, derived by LDA of the abstracts of 14,371 articles in the Neurosynth database. We visualize topic-wise surface maps that represent the cortical regions involved with a given topic. Then, in the final step of the systems-level decoding, we calculated the percentage of overlaps between the topic surface maps and the 7Networks (Yeo et al., 2011) (and vice versa) to compare our cortical systems to an established intrinsic cortical network organization of the human brain, including the dorsal attention and the frontoparietal networks, to whom the IPS has been consistently associated with. A detailed description of all analysis steps of the entire systems-level decoding is given in the Supporting information S1.

## 3. Results

### 3.1. Resting-state-functional- connectivity

We overlaid the seed-connectivity map of each IPS seed with the HCPex-MMP atlas parcellation (Huang et al., 2022) to identify cortical regions connected to the IPS regions. In the following, we will use the grouping scheme proposed by Glasser et al. (2016) who have assigned the 180 regions per hemisphere to 22 cortices (e.g., dorsal stream, …) and five cortical areas (i.e., anterior cortices, posterior cortices, early and intermediate visual cortex, sensorimotor areas, and auditory regions).

**Figure 2:**
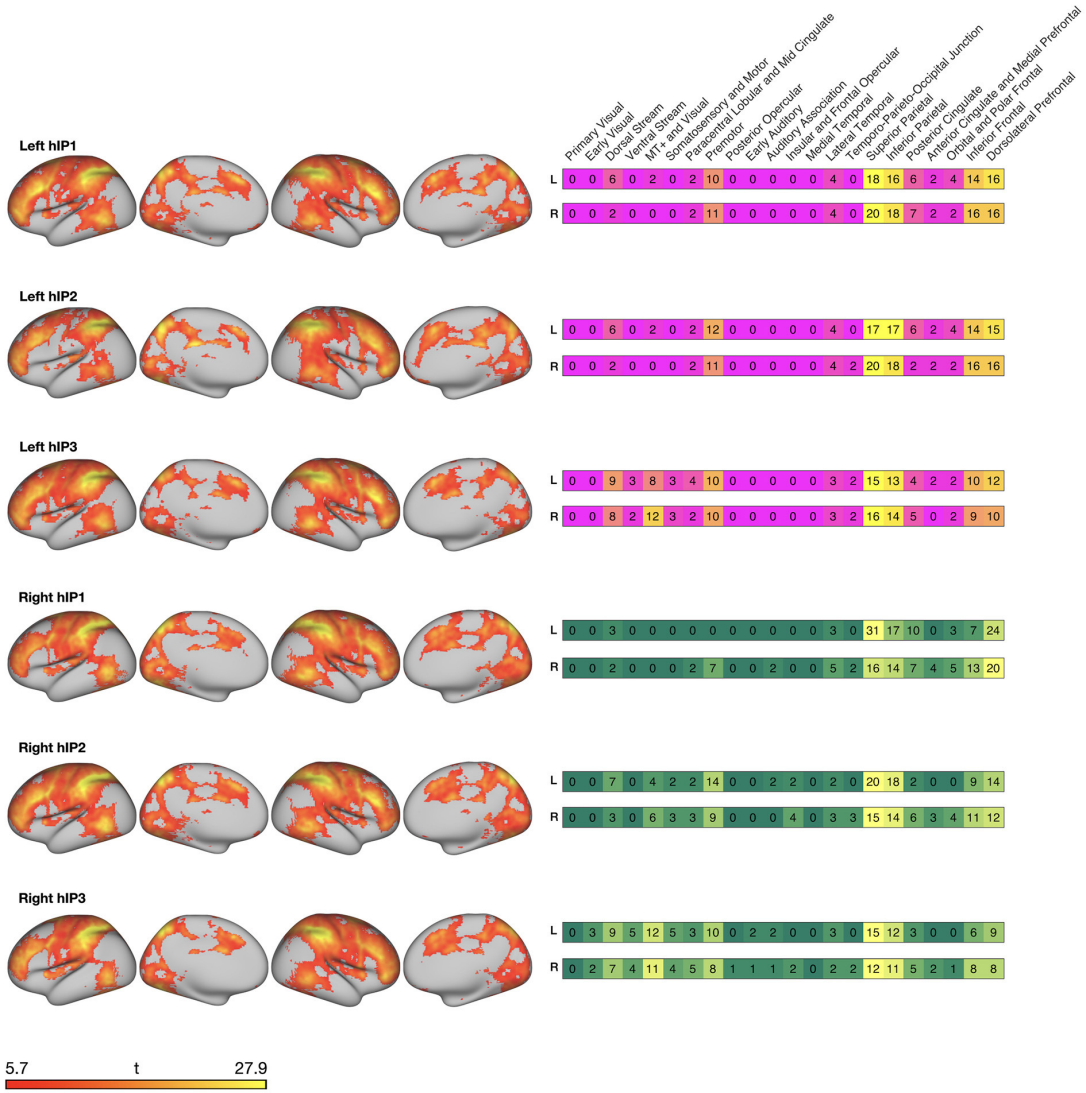
Surface maps created with connectome workbench commands (Marcus et al., 2011) displaying resting-state functional connectivity of the IPS seed regions with cortical regions. Heatmaps on the right display the relative percentage of overlap with the 22 cortices by Glasser et al. (2016).

The left hIP1 was functionally connected to 90 distinct regions. These were mainly divided over anterior, posterior, and sensorimotor areas of the cortex (see Figure 2), and smaller portions in dorsal areas of the visual cortex. Left hIP2 was also functionally connected to 90 regions. The only difference between hIP1 and hIP2 was the absence of overlap with somatosensory and motor cortices for hIP2. Left hIP3 was functionally connected with 123 regions. These regions were similar to left hIP1 and hIP2 with the main difference of a larger overlap with dorsal and ventral visual cortices of hIP3.

The right hIP1 was functionally connected to 77 regions that mainly encompassed anterior and posterior regions of the cortex. Sensorimotor areas were only connected within the ipsilateral hemisphere and functional connections with visual and temporal areas were sparse. Right hIP2 that was functionally connected to 101 regions and right hIP3 to 144 regions. For both seeds, the connected regions were divided over anterior, posterior, sensorimotor and visual areas and to a smaller extent over auditory and temporal areas of the cortex. The major difference between right hIP2 and hIP3 were a smaller engagement of visual areas for hIP2.

### 3.2. Functional characterization of IPS seed regions

**Figure 3:**
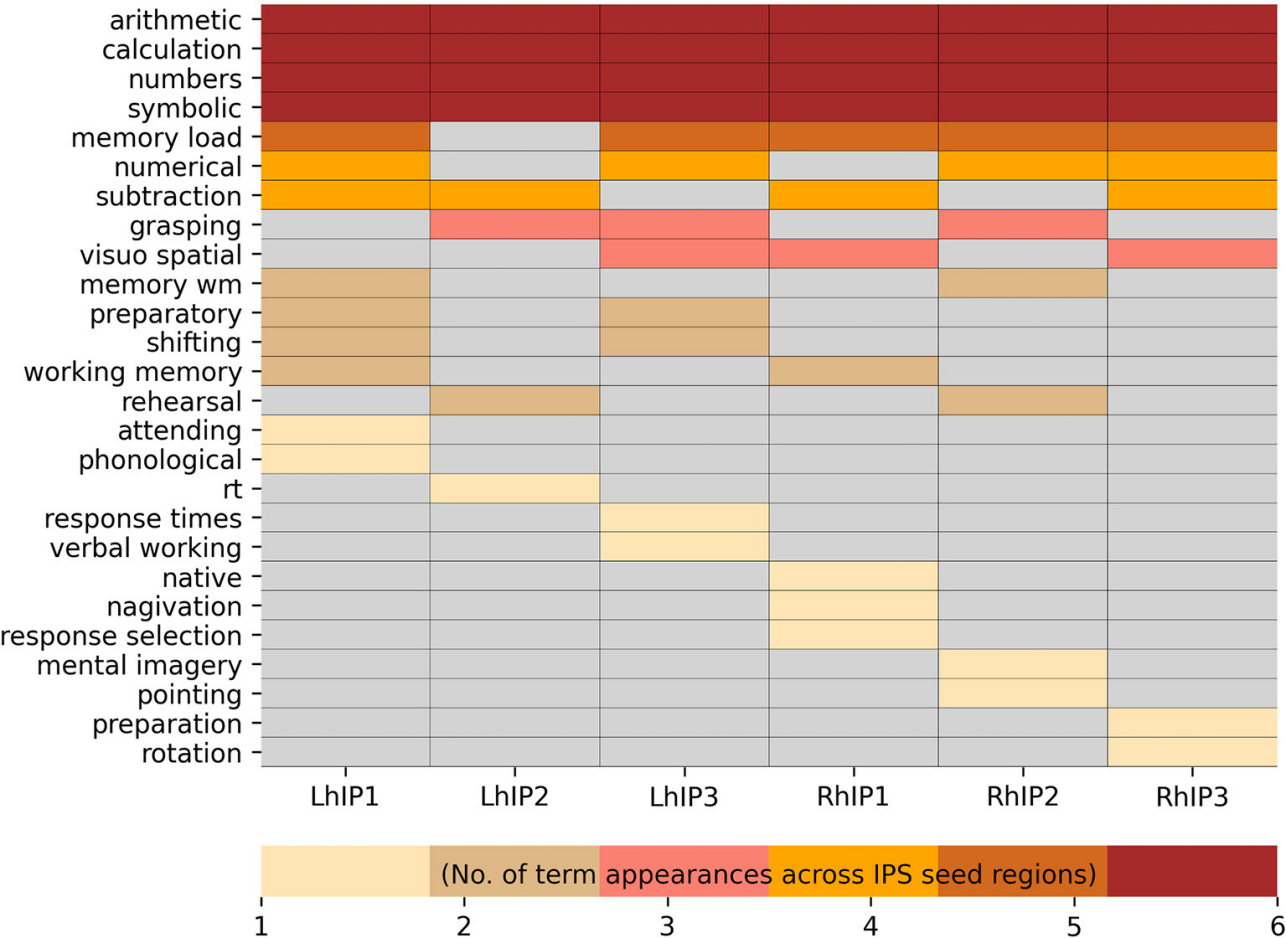
Neurosynth terms denoting psychological constructs resulting from the Bayesian reverse inference decoding summarized in a heatmap. The visualization is hierarchically ordered by the number of term appearances across the seed regions.

For each seed region, we selected the 30 decoding terms with the highest Bayesian reverse posterior probabilities, filtered to include only psychological constructs (see Supporting information S2 for a full list of all significant terms with a reverse probability over >0.05). Bayesian reverse posterior probabilities are estimates of the probability of the occurrence of a term in the database, given activation foci within a seed region. Common terms for the left hemispheric seeds (i.e., left hIP1, hIP2, and hIP3) were “arithmetic,” “symbolic,” “calculation” and “numbers.” For the right hemispheric seeds, common terms associated with all IPS seeds were “arithmetic,” “symbolic,” “numbers,” “calculation,” and “memory load.” We also found evidence for functional segregation within IPS: The decoding revealed ten terms associated with several but not all seed regions and 12 terms that were uniquely assigned to one seed region only. A full list of terms denoting psychological constructs, ordered by the frequency of their assignment to all seed regions is given in Figure 3.

### 3.3. Systems-level decoding

The aim of the systems-level decoding was to decode structure-function relationships with respect to the distinct IPS regions and their embedding into cortical systems. In the following, we summarize the results by grouping all resulting terms into larger topics (see methods) and by visualizing the IPS-centered cortical systems associated with each topic as surface maps, including a spatial comparison with the 7Networks described in Yeo et al. (2011). A full table with all terms of the systems-level decoding, and a full table with all topics containing at least one term denoting psychological constructs in the systems-level decoding is given in the Supporting informations S3, S4.

#### 3.3.1. Seed cortical topic surface maps

**Figure 4:**
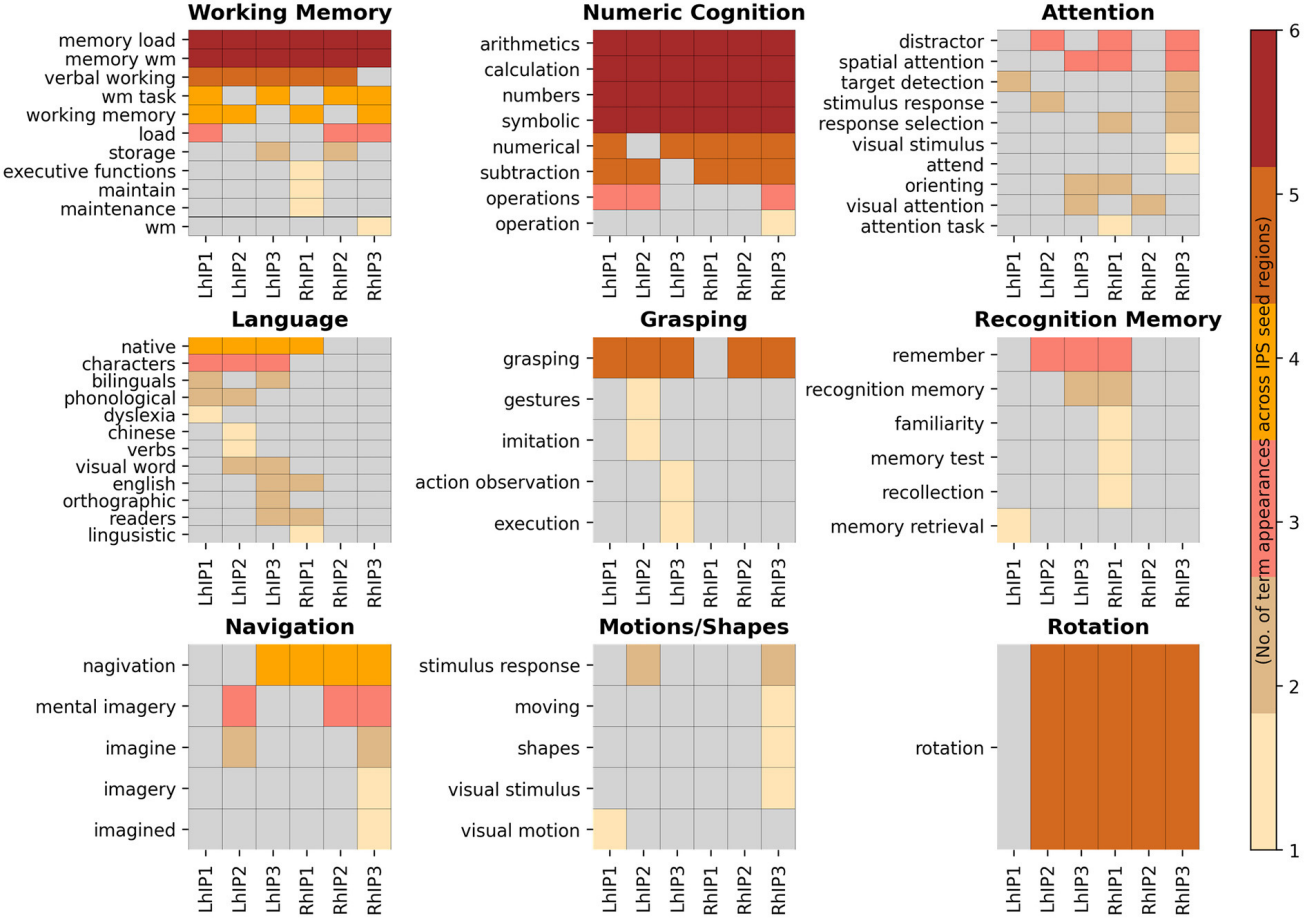
Heatmaps of the systems associated with the IPS seeds. Here, we display all systems-level decoding terms denoting psychological constructs grouped into a given topic. The visualization is hierarchically ordered by the number of term appearances across the seed regions.

**Figure 5:**
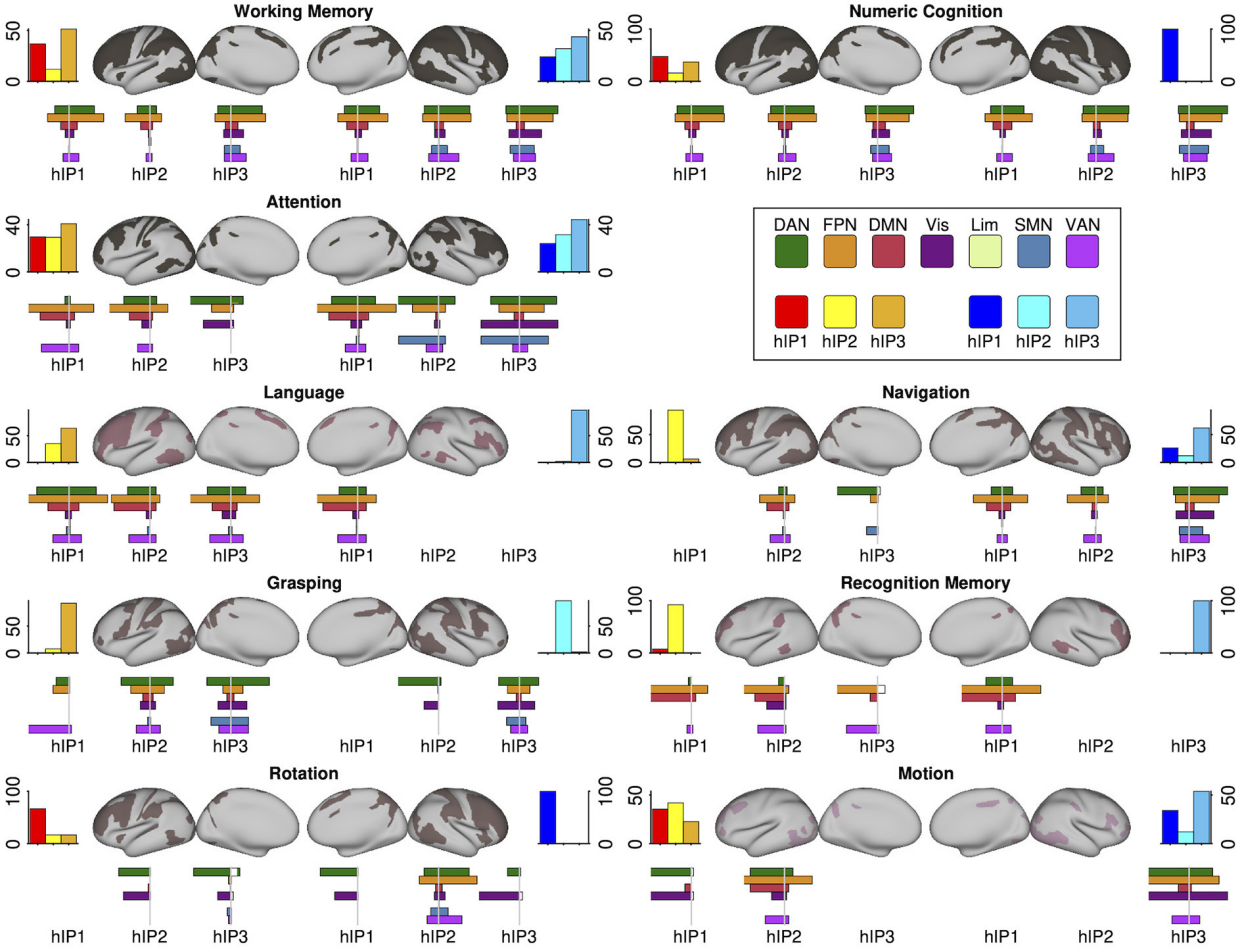
Each panel displays a cognitive/behavioral system's topology projected in an inflated cortical surface. Color gradings of the surface maps correspond the number of seed regions that build a center of a given system, with less seed regions in redder hues. The **top** bar plots flanking the surface maps to the **left and right** show the relative number of system regions per seed and hemisphere. **Below** the surface maps we present the percentage overlaps of the systems with the 7Networks separately for each seed region (plotted to the **left**). The complimentary information (percent overlap of each 7Network with the system) is plotted to the **right**. The coloring scheme for the 7Networks is the usual scheme following the original publication (see legend). Detailed statistics and precise numbers are documented in the Supporting informations S5, S6.

The systems-level decoding revealed nine cognitive systems grounded within IPS: a *working memory* and a *numeric cognition* system with connections to all three hIP seeds in both hemispheres, an *attention* system with connections to all three hIP but with preference for the right hemisphere, and six more focal systems with stronger dissociations among the three seeds and hemispheres: *language, grasping, recognition memory, rotation, detection of motions/shapes* and *navigation*. In Figure 4 we display the nine functional systems and the terms denoting psychological constructs produced by the systems-level decoding for each IPS seed that were grouped into a given topic. We present topological maps of the nine systems in Figure 5, together with information how the systems segregate across the seven canonical RSN (Yeo et al., 2011), the extent of the topological overlap with each RSN, and information on the contribution of each hIP region to the systems. A figure displaying the different contributions of every seed region within the topic surface maps is in the Supporting information S7.

The *working memory* and *numeric cognition* systems were similarly centered in all three IPS regions in both hemispheres and showed a high degree of topological overlap with each other. This was also apparent in their overlap with the 7Networks: In both systems, co-activations of each seed showed the largest overlap with the frontoparietal (FPN) and the dorsal attention network (DAN), and were absent for the limbic network. Overlap with the ventral attention network (VAN) followed a gradient across seed regions in both hemispheres, with increasing overlap from hIP1 to hIP3. Overlaps with the default mode network (DMN) were largest for bilateral hIP1, and overlap with the visual and somatomotor networks was most apparent for bilateral hIP3. The comparison with the 7Networks also revealed differences between the two systems, indicating that they are not isomorphic: The *numeric cognition* system showed relatively more overlap with the DAN relative to the FPN, the gradient of VAN contributions across seed regions was more pronounced in the *working memory* system, and right IPS showed more co-activations with the SMN in *numeric cognition* when compared to *working memory*. The *numerical cognition* system also had higher internal consistency by having very similar associations with the different terms across seed regions, while different working memory terms were more distributed across seed regions (see Figure 4).

The *attention* system was lateralized, with more contributions from seed regions in the right hemisphere. Co-activations of all three seeds in both hemispheres overlapped strongly with the FPN. Differences between the seed regions were apparent regarding the visual network and SMN: overlap with the visual overlap was relatively larger for the posterior seeds (hIP2 and hIP3) and overlap with the SMN was only visible for the posterior seeds in the right hemisphere. While all seed regions co-activated with regions within the DAN and the VAN, DAN associations were more pronounced for right hemispheric seeds and VAN associations were more pronounced in the left. Within the left hemisphere, the overlap with DAN and VAN shifted across seed regions: posterior seeds showed more DAN overlap, anterior seeds more overlap with the VAN. The terms were heterogeneously distributed over the IPS seed regions, with right hIP3 as having the largest number of term associations. Interestingly, the terms describe mostly spatial and executive (e.g., “spatial attention,” “visual attention,” “response selection,” “orienting”) processes and do not cover aspects of attentional alerting.

The IPS-centered *language* system displayed a strong lateralization toward the left hemisphere. We only observed right hemispheric contributions from hIP1. The terms building the corpus of this system can be mainly assigned to second language learning and bilingualism (e.g., “native,” “bilinguals,” “Chinese,” “English”), however, with additional terms describing visual and phonological related language processes (see Figure 4). The overlaps with the 7Networks were similar for all four implicated seed regions: The largest overlap was with the FPN, followed by DAN, VAN, and DMN. The *language* system showed only negligible overlaps with the visual and limbic networks and the SMN.

The IPS-centered *grasping* system was also more lateralized to the left hemisphere but more centered on posterior seeds. Co-activations of the three main contributing seed regions (left hIP2 and hIP3, right hIP3) showed similar topological overlap with the 7Networks, which was most strong for DAN, VAN, and the visual networks. Co-activations with the SMN was largely restricted to the most posterior hIP3.

The *mental rotation* system was the most focal system and only contained the term “rotation,” while all IPS seeds (except for left hIP1) contributed to the system, the most pronounced contribution was observed for right hIP2. The co-activations of this region were also more distributed across the 7Networks with most co-activations in the FPN, DAN, and VAN. The other four seed regions showed only co-activations with the DAN and the visual network.

The *navigation* system was more lateralized toward the right hemisphere. Of note, the major seed regions on the right were hIP1 and hIP3, while the major seed region on left was hIP2. While the co-activations of all seed regions in the *navigation* system overlapped most dominantly with the FPN, there were also differences between the seeds with more posterior seeds showing stronger coactivations with DAN, VAN, SMN, and the visual network, and more anterior seeds co-activating more extensively with regions in the DMN.

The *recognition memory* system was one of the smallest IPS-centered systems: The system was mainly centered in right hIP1 with minor links to the three seeds on the left. Co-activations of the seed region were most dominantly in the FPN and DMN, followed by the VAN.

The other small system, the *motions/shapes* system, was mainly centered in right hIP3 an left hIP2. For both seeds, co-activations overlapped with the DAN, FPN, and VAN. Right hIP3 showed also strong coactivations with the visual network, while the co-activations with left hIP2 were also pronounced within the DMN.

## 4. Discussion

The main goal of the present study was to disentangle the functional profiles of three cytoarchitecturally distinct bilateral IPS regions through meta-analytic decoding across the neuroimaging literature in two complementary ways: We conducted a standard Bayesian reverse inference decoding on the IPS regions individually, and a systems-level decoding that reveals joint seed-cortical co-activations as retrieved from the Neurosynth database. The systems-level decoding unraveled several IPS-centered functional cortical systems involved in a variety of cognitive functions. Our decoding framework revealed both similarities and dissimilarities between the different IPS regions and the behavioral relevance of their co-activation profiles.

### 4.1. Functional characterization of the IPS seeds

The standard meta-analytic Bayesian reverse inference decoding revealed that all three seeds of both hemispheres are commonly associated with terms related to *numeric cognition*. The engagement of the IPS in these processes was consistently found and is well-described in the neuroimaging literature (Menon et al., 2000; Dehaene et al., 2003; Pinel and Dehaene, 2010; Price et al., 2018). Among the theoretical considerations about the phenomenology of numeric cognition, evidence from cognitive neuroscience suggests that the IPS subserves the visual (mental) representation, manipulation and estimation of the magnitude of a number (Dehaene et al., 2003; Liu et al., 2017; Yeo et al., 2017; Bloechle et al., 2018). Furthermore, the IPS is not only linked to symbol-quantity assessments but also to basic arithmetic operations (e.g., addition, subtraction) (Wu et al., 2009; Arsalidou and Taylor, 2011; Matejko and Ansari, 2019; Castaldi et al., 2020; Roell et al., 2021). In line with these findings, the terms associated with the IPS seeds can be either be described as arithmetic operations (e.g., “calculation”) or more abstract forms of numerical representations (i.e., “symbolic,” “numbers”).

The few studies that aimed at the disentangling of the three cytoarchitecturally distinct IPS seeds in arithmetic tasks did not detect clear dissociable activation patterns in between the seeds in different arithmetic tasks. Wu et al. (2009) showed comparable levels of activation in all IPS regions during mental arithmetics and Chang et al. (2019) were able to link arithmetic aspects of word problem-solving to all three bilateral IPS seeds. Our present results confirm these findings and support the notion that all three cytoarchitecturally distinct IPS regions contribute to numeric operations.

Several studies have posed the question, whether different aspects of numeric cognition are reflected in different levels of lateralization within the IPS. In this line of reasoning, the right IPS is supposed to be involved in format-independent (i.e., irrespective whether stimuli are Arabic numerals or an array of dots) of quantity representations, and the left IPS in symbolic (e.g., Arabic numerals) processing (Venkatraman et al., 2005; Piazza et al., 2007; Holloway and Ansari, 2010). This idea is supported by stronger left IPS activation for verbal representation of arithmetic facts (Dehaene et al., 2003). We, however, did not observe lateralization differences of the IPS in numeric cognition. All three seed regions were commonly associated either with terms such as “arithmetic” and “calculation” or “numbers” and “symbolic” (see Figure 3). However, it needs to be noted that the terms in the Neurosynth database are a product of automated parsing of the literature and may be not as concise enough to distinguish between non-symbolic and symbolic arithmetic operations.

Solving arithmetic tasks depend strongly on working memory (Matejko and Ansari, 2021), so it does not come as a surprise, that the IPS has been consistently implicated in such processes. Accordingly, all seeds were associated with terms describing working memory (e.g., “working memory,” “memory wm,” “verbal working,” “memory load”). Working memory relies on three main processes: The cognitive control over the representation of information kept online, the temporary storage and the retrieval of that information (Baddeley, 1986). It has been hypothesized that the IPS plays an important role in the in the storage aspects of working memory (Christophel et al., 2012). Damage to the right IPS leads to impaired (visual) working memory (Ferber et al., 2020) and evidence from neuroimaging in healthy individuals found stronger activation in the right IPS (McNab and Klingberg, 2008). However, as for numeric cognition, we did not find a discrepancy between the IPS seeds in left and right hemispheres. The storage of information and the visuospatial attentional shift toward the location of the stored information are often described as overlapping processes (Awh et al., 1998; Awh and Jonides, 2001; Silk et al., 2010). Furthermore, focusing the mind on particular events while ignoring possible distractors can be specified as top-down guided attention, which is a cognitive function the IPS has been consistently associated with, especially in the visuospatial domain (Corbetta et al., 1998; Corbetta and Shulman, 2002; Vossel et al., 2014). The results of the functional decoding of the individual IPS seed regions are in accordance with these findings: Left hIP1 was associated with the term “attending,” and left hIP1 and hIP3 with “shifting” and left and right hIP3 with “visuospatial.”

Other terms denoting psychological constructs in this study were less homogenous in the association with the distinct IPS seeds. The term “grasping” appeared for left hIP2 and hIP3 and right hIP2. Grasping depends on the visual representation of objects in the outside world and the precise reaching for and manipulation of that object under permanent visual control. Research in primates and humans located two different sites in the brain related to grasping: Activation in the anterior bank of the IPS (AIP) is supposed to be engaged in visually controlled hand movements, whereas activation in the caudal bank (CIP) was associated with three-dimensional surface orientation and mental representation of objects (Shikata et al., 2001, 2003; Begliomini et al., 2007; Makin et al., 2007). Notably, AIP represent a putative homolog of the human hIP1, whereas the hIP3 might serve as a homolog of the medial intraparietal area (MIP) (Caspers and Zilles, 2012). However, in the present study the term grasping did not show a significant association with neither left nor right hIP1. As for the more caudal regions hIP2 and hIP3 we found terms associated with the mental representation of visual objects such as “mental imagery” and (mental) “rotation,” which might be an indicator in favor of the aforementioned distinctive roles of the IPS regions in visually guided hand movements.

### 4.2. Systems-level decoding

With the systems-level decoding we were able to unravel two large and overlapping functional systems associated with terms in the spectrum of working memory and numeric cognition that have not been described in the literature in comparable detail. We found a large attention system with a tendency toward a lateralization to the right hemisphere that complements the discussion of the overall involvement of the IPS in attentional processes. Finally, six smaller and more heterogenous systems were detected that shed new lights on the individual contribution of the IPS seeds toward cognitive functions such as language, recognition memory and navigation.

#### 4.2.1. IPS-centered brain systems involved in working memory and numeric cognition

The systems-level decoding revealed two large functional systems–working memory and numeric cognition with similar cortical topology and little differentiation between the cytoarchitecturally distinct IPS-seed regions. Both the working memory system and the system involved in numeric cognition display large overlaps with the frontoparietal and the dorsal attention network and have a similar involvement in the visual network through left and right hIP3.

The large overlap between the two systems is not surprising given the detailed evidence on the interplay between working memory and numeric cognition. Working memory predicts mathematical abilities across the lifespan (Bull and Scerif, 2001; Friso-van den Bos et al., 2013; Allen et al., 2021; Nelwan et al., 2022), and decreased working memory performances has been linked to difficulties solving mathematical problems (Swanson and Jerman, 2006; Andersson, 2008; Zhang et al., 2018). Furthermore, performing different kinds of arithmetic tasks (e.g., calculation and word-problem solving) rely on several cognitive subsystems including working memory (DeStefano and LeFevre, 2004; Raghubar et al., 2010; Cragg et al., 2017). Solving a multidigit calculation, for instance, requires the processing of the task at hand, the storage of intermediate steps of the calculation, and the manipulation of this kind of information in mind (DeStefano and LeFevre, 2004; Peng et al., 2016). Further evidence, for the pivotal role of working memory in numeric cognition comes from the neuroimaging literature, which documents overlapping activations during arithmetic and working memory tasks (Owen et al., 2005; Arsalidou and Taylor, 2011; Rottschy et al., 2012; Fedorenko et al., 2013; Matejko and Ansari, 2021). These overlapping regions build a frontoparietal circuit that includes the IPS, the supramarginal gyrus, premotor cortex and the lateral prefrontal cortex (Menon, 2016), which are a subset of the regions that we uncovered through he systems-level decoding.

Both systems showed nearly identical patterns of overlaps with the frontoparietal DAN and the FPN across both hemispheres. While both ICNs feature the IPS as putative hub (Fox et al., 2005; Dosenbach et al., 2007; Power et al., 2011), previous work has rather focused on functional differences between the two networks (Vincent et al., 2008; Spreng et al., 2010; Fedorenko et al., 2013; Cole et al., 2014), for example in regard to attention (Corbetta and Shulman, 2002; Corbetta et al., 2008; Vossel et al., 2014; Markett et al., 2022), cognitive control (Cole and Schneider, 2007; Power et al., 2011; Marek and Dosenbach, 2018), cognitive flexibility (Cohen et al., 2014), but also to working memory and numeric cognition (Champod and Petrides, 2010; Fedorenko et al., 2013; Markett et al., 2018; Chang et al., 2019). In this light, the almost identical coactivations pattern of the IPS with both ICN during working memory and numeric cognition suggests more similarity between the two ICN than previously thought.

Another goal of this study was to disentangle the role of the cytoarchitectural distinct IPS region regarding their functional roles. In contrast to the standard decoding, where we found subtle differences between the seed regions, and mainly bilateral hIP1 to be associated with the terms “working memory” and “memory wm,” the systems-level decoding revealed associations with the term “memory wm” for all seed regions. This discrepancy suggests a direct involvement of hIP1 in working memory while hIP2 and hIP3 participate in working memory through their co-activation with other cortical areas. An examination of the distinct roles of the IPS seeds in cognition, also includes an examination of differences between the hemispheres. We did not find signs of lateralization in working memory & numeric cognition, which matches the results from the standard decoding but does not align with previous studies on frontoparietal networks (Matejko and Ansari, 2021) or individual brain regions in working memory and/or numeric cognition (Dehaene et al., 2003; Piazza et al., 2007). While we found an engagement of all three IPS regions in both systems, and thus little evidence for dissociating roles of different areas along the IPS, there were subtle differences that still suggest different roles of IPS subregions. First, regions in the DMN showed a preference of overlaps with bilateral hIP1. Second, the bilateral hIP3 showed greater overlap with the visual network. This finding is consistent with earlier attempts in dissociating the connectivity of the three IPS seeds, where the connectivity between the hIP3 and occipital regions were found to be greater in extent than for hIP1 and hIP2 (Uddin et al., 2010; Price et al., 2018) and where the posterior caudal IPS is jointly involved with visual areas in the processing of three-dimensional information (Jastorff et al., 2016; Welchman, 2016). The latter and the fact that this difference in connectivity is not equivalent in respect to the terms that build the corpus of the two functional IPS centered systems (see Figure 4), might be in line with earlier hypotheses stating that the hIP3 is involved in spatial representation of both format-independent quantity and symbolic numerical representations [cf., (Uddin et al., 2010)].

#### 4.2.2. IPS-centered attention system

The IPS is well-known for its role in multimodal attention, particularly through its role as a putative hub in the DAN. Previous research suggests that the DAN is involved in the top-down goal-directed attentional selection based on internal goals or expectations (Corbetta et al., 1998, 2008; Corbetta and Shulman, 2002; Fox et al., 2005). In line with this work, we found strong meta-analytical associations between attention and coactivations of the IPS with large parts of the DAN. Previous work also suggests that the DAN interacts dynamically with regions in ventral frontoparietal areas that form a functional brain system labeled as ventral attention network (Corbetta et al., 2008), salience network (Seeley et al., 2007) or as cingulo-opercular network (Dosenbach et al., 2008). We observed attention-related coactivations between the IPS and the VAN, but these coactivations were less widespread than the coactivations with the DAN. Across different IPS regions, we also observed a negative relationship between DAN- and VAN-coactivations: Particularly in the left hemisphere, co-activations fell either in the DAN or in the VAN. This would be in line with the proposed dissociation of the two ICN in attention and their complex interplay during top-down and bottom-up attention (Katsuki and Constantinidis, 2014; Vossel et al., 2014; Suo et al., 2021). Previous work also suggests a dissociation between the FPN and the DAN during attention: The FPN is thought to be involved in executive attentional control while the DAN is involved in the spatial orienting of attention (Petersen and Posner, 2012). We, however, observed a similar degree of co-activation of both networks with IPS regions during attention. On the term-level, we also found associations that suggest an involvement of the IPS-centered attention system in both spatial orienting and selective executive attention. These findings support previous suggestions that the FPN interacts closely with the DAN during visuospatial attention (Spreng et al., 2010; Dixon et al., 2017, 2018) and that different attention functions dissociate less across ICN than previously thought (Markett et al., 2022).

Only few works have addressed the role of different IPS divisions in attention. We observed stronger co-activation of posterior hIP3 with visual regions and stronger co-activations of hIP1 with the default mode network. This fits into previous reports on within-IPS differentiations (Gillebert et al., 2013) and supports the idea that the attention system is distinct from the sensory (visual) areas that it modulates (Posner and Dehaene, 1994). A more direct link between the default mode network and visual processing and attention has also been suggested previously (Szinte and Knapen, 2020; Markett et al., 2022). We also found the attention system to be lateralized toward the right hemisphere, which fits into the neuropsychological literature on attention deficits after right-hemispheric lesions (Corbetta and Shulman, 2011; Vossel et al., 2014).

#### 4.2.3. Heterogenous IPS-centered functional systems

The IPS-centered *language* system displayed a lateralization toward the left hemisphere, in line with this hemisphere's dominance in language functioning. We found overlaps between the left IPS seeds and the right hIP1 and the FPN, the DAN and the VAN. The terms building the corpus of this system can be mainly assigned to second language learning (e.g., “native,” “bilinguals,” “Chinese,” “English”). This fits previous work that implicated the left intraparietal lobule (i.e., the angular gyrus) in language learning (Della Rosa et al., 2012; Barbeau et al., 2017) and highlighted a language control network consisting of anterior cingulate/pre-SMA, thalamus, basal ganglia, left intraparietal lobule (i.e., left angular gyrus) and frontal control regions (i.e., left prefrontal cortex) in switching between multiple languages (Abutalebi et al., 2013; Abutalebi and Green, 2016; Yuan, 2021). The results of the systems-level decoding suggests equally important involvement of the three left IPS subregions, presumably through the control of eye movements accomplished by DAN and VAN components (Corbetta et al., 1998, 2008; Corbetta and Shulman, 2002; Henderson et al., 2018), if language stimuli are presented visually (e.g., reading) as done in many natural language tasks.

A similar tendency toward lateralization to the left hemisphere was existent for the *grasping* system we found in the present study. Grasping requires the visually guided control of arm or hand movements, associated with frontoparietal areas (Binkofski et al., 1999, 2016; Grefkes and Fink, 2005; Filimon, 2010; Cavina-Pratesi et al., 2018) which might explain the large overlaps of this system with the DAN, the VAN and FPN. Neurons in the caudal bank of the macaque brain react to visual and somatosensory stimuli (Matelli and Luppino, 2001), which might serve as a further explanation for the large overlaps with the visual network and SMN in the left hIP3 that presents a putative homolog of the macaque MIP (Caspers and Zilles, 2012). The results in the present study represent, however, a viable starting point for future work, examining the specialized role of cytoarchitectural distinct IPS seeds in grasping.

By contrast, the IPS-centered system involved in *navigation* showed a lateralization to the right hemisphere with a peak (i.e., in respect to the spatial extent) on right hIP3. This right hemispheric dominance was accompanied by substantial overlaps with the VAN, the FPN, the DAN, visual areas and the SMN. Spatial navigation depends on the integration of exogenous sensory (i.e., especially visual) information and a controlled course of action if a visual cue is behaviorally relevant. Both actions are accomplished by the interactions of brain regions located in the VAN, the DAN and the FPN (Corbetta and Shulman, 2002; Corbetta et al., 2008; Chica et al., 2014), with a hypothesized right lateralization of the VAN as found in the present study.

Another system with the largest spatial extent on right hIP3, is involved with the detection of objects in the environment based on *motions and shapes*, which aligns with assumptions about the role of the IPS in object recognition [for reviews see Grefkes and Fink, 2005; Binkofski et al., 2016]. The detection of objects in the environment depends heavily on the processing of visual information, the maintenance of this information in visual short-term memory, attending to the stored information and the selection of an adequate response, accomplished by the interaction of distributed brain areas in prefrontal cortex, visual areas and the IPS (Xu and Chun, 2006; Xu, 2009; Harrison et al., 2010; Christophel et al., 2012; Xu and Jeong, 2015). The overlaps of the right hIP3-centered system components in this study with the visual network, the DAN & FPN and of the left hIP2 components with the FPN are thus in line with recent work. However, we found the left hIP2 components to be associated with the term “stimulus response” which presents a cognitive process which is not exclusively tied to the detection of motions and shapes. Moreover, the left hIP1 components were associated with “visual motion” but showed almost no significant overlaps with any of the 7Networks. Thus, further research is needed to elaborate on the dissociation between left hIP and right hIP3 as found in the present study.

Interestingly, except from left hIP1 all seed regions were associated with the term “rotation,” while the respective IPS-centered system is mainly based on right hIP2 displaying large overlaps with the DAN, the FPN, the VAN and the visual and the somatomotor network. Activation during mental rotation was robustly found in frontoparietal areas including the IPS (Podzebenko et al., 2005; Weiss et al., 2009). Studies aiming at a differentiation of the IPS seeds as used in our study, found stronger connectivity between hIP3 and visual areas (Uddin et al., 2010; Price et al., 2018), while previous work also highlighted the role of this subdivision in the manipulation of visual information (Papadopoulos et al., 2018) and visuospatial attention (Gillebert et al., 2013), which aligns with the general findings presented here.

Finally, the *recognition memory* system centered on right hIP1 (with some limitations also on the left hIP1) consisted of regions of the FPN, the DAN, the DMN and VAN. Notably, the IPS has been consistently associated with familiarity-related processes of memory recollection (Wagner et al., 2005; Yonelinas, 2005; Berryhill et al., 2007; Johnson et al., 2013). Allocating resources to the recollection of a memory are initiated by top-down guided attentional control processes involving regions in the DAN, while activation in the VAN reflects the bottom-up sensory guided pull of attention induced by the retrieved memory (Cabeza et al., 2008; Ciaramelli et al., 2008; Hutchinson et al., 2014; Monge et al., 2018). Finally, activation in the DMN have as well be associated with episodic memory retrieval (Spreng et al., 2010).

Notably, common to all systems presented here, is a strong engagement of cortical areas in either/or the DAN, FPN and the VAN. We therefore propose that the IPS plays a domain-general role in mediating between these systems, for instance, by allocating attentional resources in interaction with frontal executive areas. The systems-level decoding also unraveled differences in between the participation of the different seed regions in cortical functioning, which might serve as a promising lead for future studies. For instance, we found stronger co-activations of visual and sensorimotor areas with hIP3 seed regions in working memory, numeric cognition, attention, grasping, navigation and in the detection of motions and shapes in comparison to hIP1 and hIP2, while the association with (e.g.,) recollection memory seems to be restricted to a single IPS seed (i.e., bilateral hIP1).

### 4.3. Conclusion

The novel systems-level decoding approach proved useful in recovering structure-function associations of the IPS beyond the localized associations from the standard Bayesian reverse decoding framework. The cognitive profile of the IPS is heterogeneous but also quite uniform across different subregions. Not surprisingly for a putative cortical hub region, the IPS's functional repertoire becomes most visible in conjunction with its functional interactions with widespread cortical regions across several intrinsic networks. The present results provide a reference for the interpretation of cognitive neuroscience experiments and highlights several starting points for future investigations for the connectome-wide representation of cognitive and behavioral functions.

## Data availability statement

The raw data supporting the conclusions of this article will be made available by the authors, without undue reservation.

## Ethics statement

All analysis used secondary data and no new human participants were generated. Ethics approval was obtained by the authors of the original datasets from their respective ethics review boards.

## Author contributions

Material preparation and data collection was performed by OB and SM. OB implemented the computer code, supporting algorithms, and analyzed the data. OB drafted the manuscript and final revisions were done by SM. All authors contributed to the study conception, design, read, and approved the final manuscript.
