# Supplementary material for: Systems-level decoding reveals the cognitive and behavioral profile of the human intraparietal sulcus
Source: Front Neuroimaging. 2023 Jan 9;1:1074674. doi: 10.3389/fnimg.2022.1074674 (PMC10406318; doi:10.3389/fnimg.2022.1074674)
Supplement: Supplementary file 1 [file Data_Sheet_1.pdf]

Supplementary Material

Supporting Information 1: Table with a detailed description of the different steps of the systems-level decoding.

Step No.:	Description:	Purpose:
1. First iteration of the decoding: Neurosynth database query with seed-cortical pairs	Generate masks consisting of the seed region and the cortical region connected to that seed region at rest. Repeat for all connected regions. Enter all seed-cortical pairs into the Neurosynth Decoder for the database query.	Aggregate the data of the systems-level decoding into a large dataset.
2. Aggregation of top30 terms of each seed-cortical pair	Store the top30 terms (i.e., based on the posterior probability) of each co-activated seed-cortical pair in a large dataset.	
3. Data cleansing – Exclude non-significant terms	Exclude all terms, that did not pass the multiple comparison correction from the same dataset (i.e., < pReverse 0.05).	
4. ID-tagging of the terms of the database query	Tag each term in the dataset with the respective HCPex regional ID of the cortical region that produced the term in conjunction with the given seed region.	This step of the analysis is done to enable, in a later process of the data analysis, the identification of all cortical regions that are associated with the occurrence of a given term.
5. Second Iteration of the decoding: <br>Neurosynth database query with the cortical regions only.	Second iteration of the decoding, this time solely for the cortical regions (i.e., without the seed region) with applying the same data aggregation steps to the results, as applied to the seed-cortical pairs (see steps 1-4). This results in the accumulation of all filtered and "ID-tagged" terms in a second dataset.	At this point of the analysis, the reason for the occurrence of a term might be threefold: 1. The term occurs in studies that report IPS-seed activation. 2. The term occurs in studies that report activation in the co-activated cortical region. 3. The term occurs in studies that report activation with the seed region and the co-activated cortical region. Thus, the second iteration and steps 6-7 of the decoding are done to exclude an
6. Data cleansing – Set intersections	Calculate the *set intersection* between the dataset for the seed-cortical pairs and the	

	dataset of terms from the cortical regions with a *logical-&-conjunction.*<br><br>This step of the analysis produces a list of terms that exist in both datasets with no repetitions.	influence above chance of the second scenario.
7. Data cleansing – Set differences	Use the regional IDs all terms were tagged with, to calculate the *set difference* between the IDs of the first iteration (i.e., the decoding of the seed-cortical pairs) and the second iteration of the decoding (i.e., the decoding of the cortical regions), which returns all terms tagged with an ID that are not present in the set of terms of the second iteration of the decoding with no repetitions.	
8. Data refinement: Grouping of the terms into larger Neurosynth Topics	Group the terms into 50 Topics as provided on the Neurosynth webpage, by simply testing whether a given term is listed in the topic or not. However, within the 50 topics, terms are presented as single terms (e.g., memory) whereas the Neurosynth corpus can consist of compound terms (e.g., 'working memory'). Therefore, split the compound terms into two sub-components (i.e., 'working', 'memory') and only grouped the original term into a topic, if both term components are listed in the respective topic.	
9. Data visualization: create topic surface maps	Merge the cortical ID's of each term that belongs to a particular topic, so that surface maps can be generated that represent a cortical system involved with a given topic. Surface maps are generated with the connectome workbench wb_commands (Marcus et al., 2011).	Visualization of the data.
10. Data refinement: compute overlaps with the 7Networks (and vice versa)	Calculate percentage of overlaps between the topic surface maps with the 7Networks (Yeo et al., 2011) in two ways: First, computed the overlaps as the number of voxels for a given network (i.e., the dorsal attention network), that overlaps with the voxels in the topic surface map divided by the total number of voxels in the given network. Second, compute the percentage of voxels in the topic surface map that lie within a given network.	Situate the topic surface maps within cortical intrinsic resting state networks.

References:

Marcus, D. S., Harwell, J., Olsen, T., Hodge, M., Glasser, M. F., Prior, F., et al. (2011). Informatics and Data Mining Tools and Strategies for the Human Connectome Project. Front. Neuroinformatics 5. doi: 10.3389/fninf.2011.00004.

Yeo, B. T., Krienen, F. M., Sepulcre, J., Sabuncu, M. R., Lashkari, D., Hollinshead, M., et al. (2011). The organization of the human cerebral cortex estimated by intrinsic functional connectivity. *Journal of Neurophysiology* 106, 1125–1165. doi: 10.1152/jn.00338.2011.

Supporting Information 2: Table of all terms with a sign. level p.<0.05 and a reverse probability of *probReverse above* >0.5 of the Bayesian reverse inference decoding sorted by the strength of the reverse probability in descending order. Top30 terms are highlighted in green. Psychological constructs from the top30 terms, that were selected for interpretation are highlighted in dark green.

Seed Region	Terms	pReverse	probReverse
LhIP1	calculation	8,78E+05	0.76
	symbolic	1,18E+10	0.74
	numbers	1,08E+07	0.74
	intraparietal	9,79E-32	0.73
	intraparietal sulcus	1,15E-23	0.73
	sulcus ips	2,92E+05	0.72
	arithmetic	2,43E+10	0.71
	subtraction	2,43E+10	0.71
	ips	9,25E+04	0.71
	anterior intraparietal	0.000133046290623	0.71
	cortex ppc	7,41E+09	0.70
	memory load	0.0002273528394328	0.70
	reliance	0.0002273528394328	0.70
	ppc	5,52E+11	0.70
	preparatory	0.0001706765437168	0.70
	frontoparietal network	2,15E+11	0.70
	frontoparietal	4,12E+00	0.70

	numerical	4,67E+10	0.70
	parietal network	2,38E+07	0.69
	attention network	1,18E+09	0.69
	flexibly	0.0036826328952178	0.69
	memory wm	1,11E+06	0.69
	posterior parietal	8,23E-02	0.69
	shifting	0.0001706765437168	0.69
	parietal	1,42E-46	0.68
	attending	0.0068025826432386	0.68
	phonological	1,05E+05	0.68
	working memory	2,06E-13	0.68
	prefrontal parietal	4,03E+11	0.68
	eye field	0.0029564725931788	0.68
	behavioral level	0.0055336351444703	0.68
	parietal cortices	2,56E+08	0.68
	pre supplementary	0.0003376315715421	0.68
	working	1,32E-12	0.68
	switching	6,69E+09	0.68
	visuo spatial	0.0129205244772073	0.68
	native	0.000144040549842	0.67
	parietal cortex	2,18E-11	0.67
	successive	0.013703640025737	0.67
	negative feedback	0.0182992240127633	0.67

	behavioral evidence	0.0242151759862441	0.67
	inferior superior	0.0122529619882858	0.67
	characters	0.0082328812332652	0.67
	operations	0.0001093973422107	0.67
	load	2,84E+06	0.67
	domain general	0.0108861497048898	0.67
	fronto parietal	2,95E+05	0.67
	parietal lobules	0.0189571518470182	0.66
	attentional control	0.0012427752413418	0.66
	memory retrieval	2,08E+11	0.66
	opercular	0.0193814439455	0.66
	dyslexia	0.0367847998609948	0.66
	target detection	0.0367847998609948	0.66
	response times	0.0254963048537013	0.66
	cortical networks	0.0101837390217256	0.66
	verbal working	0.0149627375049219	0.66
	task involving	0.0328564668890545	0.66
	inferior parietal	2,87E-05	0.66
	exact	0.0102147728782773	0.66
	ventral premotor	0.000602260193285	0.65
	upcoming	0.0289891008437192	0.65
	switch	0.0057449716676519	0.65
	resources	3,95E+09	0.65
	wm task	0.0289891008437192	0.65

	frontal eye	0.0019817777339456	0.65
	rules	0.0075890699188503	0.65
	memory processes	0.0254963048537013	0.65
	parietal frontal	0.0056452196098527	0.65
	wm	4,20E+09	0.65
	recollection	0.0049617251805746	0.65
	control processes	0.0049617251805746	0.65
	flexibility	0.0367847998609948	0.65
	frontal parietal	4,23E+08	0.64
	color	0.00033760945764	0.64
	interference	1,63E+09	0.64
	requirements	0.0455902365510952	0.64
	discrimination task	0.0315211822377134	0.64
	memory tasks	0.0209356495745234	0.64
	pitch	0.0449348015126686	0.64
	likelihood	0.039571678249439	0.64
	speakers	0.0342043897367073	0.64
	familiarity	0.0049157514394778	0.64
	maintenance	3,93E+10	0.64
	overlapped	0.033196590451173	0.64
	comprised	0.0289891008437192	0.64
	learned	6,56E+10	0.63
	selectively	6,29E+10	0.63

	rule	0.0128948595340559	0.63
	verb	0.0438009770199624	0.63
	success	0.0119340241768676	0.63
	absent	0.0423525492042958	0.63
	rely	0.0013303498317617	0.63
	domains	0.0027692258429241	0.63
	version	0.0075464462609122	0.63
	competing	0.0494591752077207	0.63
	flexible	0.0374037664817924	0.62
	visuospatial	0.0028461726676791	0.62
	executive control	0.0068025826432386	0.62
	correct	0.0003820737825905	0.62
	lateral prefrontal	0.0003820737825905	0.62
	parietal lobule	1,49E+09	0.62
	engagement	0.0005473406772274	0.62
	memory	1,57E-05	0.62
	memory task	0.0003020814639487	0.62
	sulcus	9,90E+03	0.62
	recognition memory	0.0493494757911583	0.62
	similarity	0.0199029704295976	0.62
	tasks	3,38E-02	0.62
	fronto	2,08E+08	0.62
	demands	6,80E+08	0.62
	parieto	0.0249707864222533	0.62

	distance	0.0454024669608562	0.62
	reasoning	0.0321709428227249	0.62
	superior parietal	6,44E+09	0.62
	planning	0.0078993628974832	0.62
	dissociable	0.0270148414939847	0.62
	task relevant	0.0367847998609948	0.62
	domain	0.0006706464903098	0.62
	preparation	0.0495638010960535	0.62
	judgment	0.0069647428900816	0.61
	cognitive processes	0.0062103516482467	0.61
	item	0.0110225529956676	0.61
	episodic	0.0002443193377006	0.61
	term memory	0.034694256240382	0.61
	multivariate	0.008368929806638	0.61
	idea	0.0247698337738531	0.61
	predictions	0.0379235171857831	0.61
	engage	0.0110225529956676	0.61
	temporo parietal	0.0175136963590792	0.61
	difficulty	0.0018593508691744	0.61
	irrelevant	0.0254963048537013	0.61
	read	0.0473418101605946	0.61
	shift	0.0187151498934294	0.61
	items	0.0014349687193068	0.61

	retrieval	4,20E+09	0.61
	pairs	0.0078993628974832	0.61
	relies	0.0391664823077383	0.61
	capacity	0.0207848377321648	0.60
	parietal lobe	0.0086725065346482	0.60
	attentional	2,37E+10	0.60
	lobule	2,63E+10	0.60
	dorsolateral	2,08E+08	0.60
	recruited	5,07E+11	0.60
	engaged	7,32E+10	0.60
	set	0.0001198088548245	0.60
	ventrolateral prefrontal	0.0240300624259787	0.60
	cortex inferior	0.0473418101605946	0.60
	modality	0.0119340241768676	0.60
	modalities	0.0342043897367073	0.60
	reading	0.0030872340044475	0.60
	sequences	0.0401248211572574	0.60
	temporo	0.0209356495745234	0.60
	abstract	0.0427425208600354	0.60
	episodic memory	0.0301195913163309	0.59
	visually	0.0319318411185994	0.59
	linguistic	0.0379516510766111	0.59
	manipulation	0.0423829605438032	0.59
	active	0.0001973559324223	0.59

	varying	0.039571678249439	0.59
	eye	0.0199934959539978	0.59
	overlapping	0.0274234443569376	0.59
	strategies	0.0367847998609948	0.59
	execution	0.0288220683111597	0.59
	ventrolateral	0.015850939686227	0.59
	dorsolateral prefrontal	0.0001034946812536	0.59
	differentially	0.0361399527975047	0.59
	monitoring	0.0178068012330975	0.59
	word	0.0002962779752148	0.59
	executive	0.0011967711039027	0.59
	task	4,27E+03	0.59
	network	4,24E+05	0.59
	selection	0.0212722567650079	0.59
	processes	3,77E+08	0.58
	task performance	0.0210015474240024	0.58
	extent	0.0039539437512316	0.58
	required	0.004990861578119	0.58
	location	0.0233031642228916	0.58
	general	0.0008368241464673	0.58
	attention	9,33E+09	0.58
	conclude	0.0438009770199624	0.58
	inferior	2,63E+07	0.58

	event functional	0.0345646941654166	0.58
	premotor cortex	0.029113235481301	0.58
	target	0.0110225529956676	0.57
	learning	0.0027924654382701	0.57
	cues	0.0197950121305598	0.57
	involvement	0.0149627375049219	0.57
	common	0.005299948466005	0.57
	cognitive control	0.0473045662127494	0.57
	representations	0.0133621650949374	0.57
	spatial	0.001306288895919	0.57
	object	0.0187151498934294	0.57
	words	0.0131152152046194	0.57
	number	0.0176574109947597	0.57
	supplementary	0.0237997945237369	0.57
	networks	0.0002880773456959	0.57
	event	0.001856679789019	0.57
	premotor	0.0152491241856685	0.57
	information	0.0001223284442882	0.56
	second	0.0302336605157785	0.56
	performance	0.0001378409228782	0.56
	semantic	0.0434791364295235	0.56
	frontal	8,10E+10	0.56
	language	0.0434791364295235	0.56
	conditions	0.0103298892469087	0.56

	involved	0.0007795567328755	0.55
	activations	0.0274234443569376	0.55
	contrast	0.0153271464318294	0.55
	adults	0.0420616685417192	0.55
	cognitive	0.0075464462609122	0.55
	visual	0.0110225529956676	0.55
	posterior	0.0493012971819757	0.54
	prefrontal	0.0356106340057453	0.54
LhIP2	anterior intraparietal	1,47E+04	0.80
	parietal lobules	2,61E+08	0.77
	symbolic	4,40E+09	0.76
	rehearsal	5,88E+10	0.75
	calculation	0.000102274378934	0.75
	subtraction	1,53E+11	0.74
	numbers	2,43E+10	0.74
	prefrontal parietal	4,91E+07	0.74
	successive	0.0003891361424665	0.74
	arithmetic	9,01E+10	0.73
	implementation	0.001611805379724	0.73
	requirements	0.000435362233482	0.72
	intraparietal	7,21E-08	0.72
	intraparietal sulcus	2,25E-04	0.72
	ventral premotor	1,12E+10	0.72

	lobules	8,02E+10	0.72
	lobule ipl	0.0009130365241642	0.71
	ppc	0.0033925545968941	0.71
	relied	0.0143430320430803	0.71
	expectancy	0.022806478401307	0.70
	grasping	0.0099685895137041	0.70
	ipl	0.000124022791261	0.70
	parietal	1,49E-30	0.70
	opportunity	0.0230915700663914	0.70
	parietal cortices	1,14E+11	0.69
	correspond	0.0257291109864251	0.69
	cortex parietal	0.0257291109864251	0.69
	cortex ppc	0.0198186067644361	0.69
	rt	0.0112218881769784	0.69
	parietal frontal	0.0026893711589844	0.69
	hands	0.0093809120201869	0.68
	frontoparietal network	0.0122354010768654	0.68
	sulcus ips	0.0011360672533074	0.68
	verbal working	0.031153840490504	0.68
	memory wm	5,05E+11	0.68
	posterior parietal	9,08E+06	0.68
	networks involved	0.0257291109864251	0.68
	pre sma	0.0089314363042355	0.67
	tactile	0.0004199448505254	0.67

	demands	6,00E+05	0.67
	stimulus response	0.0147420006801063	0.67
	inferior parietal	2,38E-01	0.67
	debate	0.0151940003852214	0.67
	frontal parietal	1,22E+09	0.67
	frontoparietal	1,16E+11	0.67
	task irrelevant	0.0314439325978694	0.67
	ips	0.0019946609394758	0.67
	parietal network	0.002627946308316	0.67
	distance	0.013253420060266	0.67
	fronto parietal	5,05E+09	0.66
	working memory	1,90E+03	0.66
	working	3,03E+01	0.66
	parietal cortex	1,26E+04	0.66
	operations	0.021222504276149	0.66
	mirror	0.0051147834056773	0.65
	task relevant	0.0263935822432685	0.65
	phonological	0.0003435046986565	0.65
	execution	0.0003221474981717	0.65
	parietal lobule	3,06E+08	0.65
	make	0.0024937011731352	0.65
	resources	0.002627946308316	0.64
	supramarginal gyrus	0.0051113249892968	0.64

	chinese	0.0257291109864251	0.64
	imagery	0.0038789229737879	0.64
	employed	0.0004045547315442	0.64
	wm	0.0068250221348779	0.63
	recruited	4,03E+09	0.63
	irrelevant	0.031153840490504	0.63
	tasks	5,18E+02	0.63
	supramarginal	0.0055547741439377	0.63
	sequences	0.021222504276149	0.63
	asked	0.0022072256630692	0.63
	lobule	6,94E+09	0.63
	requiring	0.0417513939488748	0.63
	domain	0.0067647890289135	0.63
	cognitive processes	0.026269410389171	0.63
	verbal	0.0001324157003939	0.62
	load	0.0190457531491676	0.62
	overlapping	0.0198186067644361	0.62
	representations	0.0002153279423513	0.61
	superior parietal	0.0035580315032801	0.61
	required	0.0020354751571907	0.61
	ba	0.0346303281668985	0.61
	involves	0.0294317529323154	0.61
	sulcus	3,76E+11	0.61
	premotor cortex	0.0095820251219522	0.61

	premotor	0.0004362706180698	0.60
	task	9,81E+04	0.60
	types	0.006172517168798	0.60
	actions	0.0245129318139696	0.60
	action	0.0051147834056773	0.60
	object	0.0107801031082495	0.59
	memory	3,78E+08	0.59
	processes	1,18E+11	0.59
	language	0.006172517168798	0.59
	common	0.0093809120201869	0.59
	inferior	4,21E+09	0.59
	mental	0.031153840490504	0.58
	fronto	0.0480754343932996	0.58
	retrieval	0.0424219598631186	0.58
	spatial	0.0136649934560938	0.58
	network	0.000102274378934	0.57
	frontal	0.0002809283198808	0.57
	suggesting	0.0441478046165088	0.57
	event	0.0285401664233732	0.57
	stimulus	0.0339449854874733	0.56
	involved	0.0035580315032801	0.56
	motor	0.0121271832759724	0.56
LhIP3	sulcus ips	1,03E-11	0.75

	intraparietal sulcus	3,92E-53	0.74
	numerical	1,04E+02	0.73
	intraparietal	4,97E-59	0.73
	ips	2,19E-10	0.73
	calculation	1,42E+07	0.73
	ppc	6,37E+03	0.73
	cortex ppc	2,61E+05	0.73
	symbolic	3,57E+09	0.71
	arithmetic	4,42E+07	0.71
	numbers	1,42E+06	0.71
	preparatory	6,43E+08	0.70
	frontoparietal network	1,12E+09	0.69
	parietal frontal	2,35E+06	0.69
	parietal network	8,80E+02	0.69
	parietal lobules	2,77E+11	0.69
	memory load	3,88E+10	0.68
	anterior intraparietal	3,06E+10	0.68
	shifting	1,78E+09	0.68
	grasping	7,82E+10	0.68
	conflicting	7,53E+09	0.68
	parietal	1,29E-72	0.68
	verbal working	3,84E+11	0.68
	fronto parietal	1,60E-03	0.67
	response times	0.0001714668599987	0.67

	superior inferior	0.0005013526816581	0.67
	inferior superior	0.0002319989083768	0.67
	cortex parietal	0.0001799564141064	0.67
	attention network	8,49E+07	0.67
	visuo spatial	0.0008308273048905	0.67
	eye fields	4,12E+10	0.67
	superior parietal	7,02E-09	0.67
	attentional control	4,07E+09	0.67
	posterior parietal	1,75E-02	0.67
	task task	0.0001155698882061	0.66
	likelihood	0.0001155698882061	0.66
	visuo	4,12E+09	0.66
	frontoparietal	5,50E+02	0.66
	saccades	0.0038313234088358	0.66
	term memory	2,06E+08	0.66
	parietal cortex	2,71E-17	0.66
	storage	0.0005520613678082	0.66
	orthographic	5,62E+10	0.66
	characters	0.0008774313103413	0.66
	visual word	0.0001957455265942	0.66
	rehearsal	0.0048600258402709	0.66
	flexibly	0.0048600258402709	0.66
	memory wm	7,24E+06	0.66

	rotation	0.0007067213043709	0.66
	execution	8,80E+02	0.65
	readers	0.0003206233782911	0.65
	stimulus driven	0.0037842725562296	0.65
	prefrontal parietal	2,55E+10	0.65
	orienting	5,12E+10	0.65
	parietal cortices	1,68E+08	0.65
	wm task	0.0017654338759892	0.65
	selective attention	8,35E+10	0.65
	allocation	0.0028720163163496	0.65
	frontal eye	2,37E+10	0.65
	computation	0.0097956346659128	0.65
	working memory	8,57E-13	0.65
	native	0.0001033459192056	0.65
	attempt	0.0028069853519847	0.65
	color	5,71E+08	0.65
	relied	0.0094813540249522	0.65
	lobules	0.0003623487788536	0.65
	interference	2,98E+07	0.65
	memory tasks	0.0006059090268982	0.65
	spatial attention	0.0001610518397009	0.65
	separated	0.0007566575874642	0.65
	english	0.0001168620561206	0.64
	working	2,19E-10	0.64

	phonological	5,90E+05	0.64
	visual attention	0.0009017629327654	0.64
	spl	0.0088840179582558	0.64
	sulcus	1,46E-11	0.64
	maintenance	5,83E+06	0.64
	visuospatial	7,52E+08	0.64
	ipl	0.0001642335443835	0.64
	rules	0.0008137533815664	0.64
	subtraction	0.006380920472799	0.64
	frontal parietal	2,20E+06	0.64
	visuomotor	0.0003607477249441	0.64
	inferior prefrontal	0.0048301761031798	0.64
	memory processes	0.0048301761031798	0.64
	switch	0.0012187218865835	0.64
	irrelevant	4,69E+09	0.64
	ventral premotor	0.0001036428100803	0.64
	bilinguals	0.0207983730738486	0.64
	recognition memory	0.0007172062032515	0.64
	resources	1,11E+09	0.64
	letters	0.0014820136590382	0.64
	parietal lobule	2,13E+01	0.64
	domain general	0.0124827574355014	0.64
	parietal lobes	0.0124827574355014	0.64

	task irrelevant	0.0025327276743543	0.64
	load	2,41E+08	0.64
	visual cortices	0.019705406471473	0.63
	retention	0.0246025613051585	0.63
	shapes	0.0013230799421586	0.63
	visually presented	0.0088664354698585	0.63
	control processes	0.0007566575874642	0.63
	upcoming	0.0146348812080441	0.63
	switching	0.0002043353381936	0.63
	expectancy	0.0368800993724385	0.63
	shifts	0.0010984823946432	0.63
	saccade	0.028329009639668	0.63
	effortful	0.028329009639668	0.63
	word form	0.0170658100643746	0.63
	remember	0.0074430001564359	0.63
	finger movements	0.0270623071263292	0.63
	shift	1,38E+10	0.63
	character	0.009344444748517	0.63
	stroop task	0.0029663127582719	0.63
	maintaining	0.0037301166537402	0.63
	implementation	0.032698060429584	0.63
	rule	0.001004032789114	0.63
	semantics	0.031270473080424	0.63
	navigation	0.0408952670954531	0.63

	planning	4,10E+10	0.63
	reading	2,49E+07	0.62
	anterior prefrontal	0.0217395154560039	0.62
	reaching	0.0217395154560039	0.62
	portions	0.0122419140623121	0.62
	cued	9,40E+10	0.62
	dorsal ventral	0.001675376947936	0.62
	discuss	0.0079058731569971	0.62
	lobule ipl	0.0192780983016253	0.62
	attention task	0.031860776835407	0.62
	motor imagery	0.0237817613496624	0.62
	solving	0.028329009639668	0.62
	stroop	0.0005019523659545	0.62
	operations	0.0028069853519847	0.62
	cortex involved	0.0268506215437049	0.62
	eye movements	0.0194979929060462	0.62
	executed	0.0342689619740796	0.62
	task difficulty	0.0064486833507483	0.62
	lobule	5,74E+03	0.62
	eye field	0.0422312400138266	0.62
	inferior parietal	2,97E+01	0.62
	task	2,90E-19	0.62
	dorsal attention	0.0395577883670634	0.62

	manipulating	0.01418014715086	0.62
	task relevant	0.0041981606400696	0.61
	wm	4,09E+10	0.61
	executive control	0.0009633736577917	0.61
	material	0.0048301761031798	0.61
	ventral dorsal	0.0318923780437383	0.61
	failed	0.0068456794235998	0.61
	visually	6,16E+09	0.61
	parametrically	0.030293790388973	0.61
	memory retrieval	0.0013878968327089	0.61
	word	9,08E+03	0.61
	tasks	7,44E-08	0.61
	fronto	8,18E+05	0.61
	coordination	0.0147304606855654	0.61
	maintained	0.0090084746810909	0.61
	item	0.0007531213398725	0.61
	flexible	0.0164729755727076	0.61
	fields	0.0056611843809736	0.61
	serial	0.0310300478025018	0.61
	complexity	0.0030678985698633	0.61
	retrieval	6,26E+05	0.61
	task performance	4,91E+09	0.61
	action observation	0.0430487586496781	0.61
	chinese	0.0043597179994971	0.61

	premotor	2,12E+05	0.61
	preparation	0.01418014715086	0.61
	correct	0.0001621238179041	0.61
	similarity	0.0081737238738155	0.61
	correctly	0.0265242181059383	0.60
	targets	0.0002653582427567	0.60
	dorsal premotor	0.0099781504989511	0.60
	stimulus response	0.0361254949778598	0.60
	attentional	1,03E+08	0.60
	languages	0.0292374329818994	0.60
	executive functions	0.0463419823650302	0.60
	require	0.0019510833572659	0.60
	manipulation	0.0011128576189923	0.60
	memory task	0.0002953279558377	0.60
	argue	0.0417822333124824	0.60
	judgments	0.0004299740396532	0.60
	letter	0.0240047007330967	0.60
	memory	2,77E-03	0.60
	sets	0.01418014715086	0.60
	abstract	0.0041981606400696	0.60
	parietal lobe	0.001231112354886	0.59
	efficient	0.0122340207203597	0.59
	difficulty	0.0007067213043709	0.59

	imagery	0.0038128796240461	0.59
	words	1,23E+09	0.59
	set	3,07E+09	0.59
	concept	0.0437816874099079	0.59
	target	7,52E+08	0.59
	eye	0.0008387389075578	0.59
	rely	0.0093435032360712	0.59
	dual	0.0241143777118312	0.59
	skills	0.0256504367004478	0.59
	younger	0.0274830699446707	0.59
	parieto	0.0363877229182416	0.59
	abilities	0.0081737238738155	0.59
	demands	7,61E+10	0.59
	engaged	4,07E+09	0.59
	orientation	0.0422428873911258	0.59
	guided	0.027742307944059	0.59
	phases	0.0487942766889082	0.59
	read	0.0341842130412563	0.59
	conclude	0.0007820887881984	0.59
	version	0.0281777098612711	0.59
	allows	0.0235131047859206	0.59
	using event	0.0255688544420672	0.59
	pairs	0.0060496796687067	0.59
	goal directed	0.035779847293115	0.59

	supramarginal gyrus	0.013157321288059	0.59
	regardless	0.0061712300589879	0.58
	encoded	0.0244676267267842	0.58
	probe	0.0481325948513897	0.58
	requiring	0.0246025613051585	0.58
	engagement	0.0078253568920305	0.58
	objects	9,39E+10	0.58
	faster	0.0278947418664381	0.58
	cue	0.0022128083341515	0.58
	conflict	0.0099119242411979	0.58
	verbal	7,61E+10	0.58
	visual	7,90E+01	0.58
	required	0.0001693889497568	0.58
	executive	5,93E+10	0.58
	learned	0.0094134702454161	0.58
	conjunction	0.0192454683823549	0.58
	domain	0.0075773334912043	0.58
	perform	0.0148036180700704	0.58
	representations	3,10E+10	0.58
	premotor cortex	0.0008910903435663	0.58
	locations	0.0437755925479101	0.58
	cognitive control	0.0011128576189923	0.58
	reaction time	0.0311246421847238	0.58

	action	8,35E+10	0.58
	generally	0.0148891352218772	0.58
	manipulated	0.0170925235197976	0.58
	judgment	0.035779847293115	0.58
	network	1,81E+03	0.58
	event functional	0.0023056171298419	0.58
	dorsolateral	3,29E+10	0.58
	shared	0.035779847293115	0.58
	supramarginal	0.0183261488827842	0.57
	lateralized	0.0241477441435117	0.57
	dorsolateral prefrontal	4,10E+10	0.57
	selection	0.0075695722998447	0.57
	lateral prefrontal	0.0241143777118312	0.57
	object	0.0002691136630392	0.57
	processes	1,45E+07	0.57
	recruitment	0.0037450100774078	0.57
	frontal	7,19E+02	0.57
	inferior	5,77E+04	0.57
	attention	2,62E+09	0.57
	modality	0.025312767625709	0.57
	requires	0.0192454683823549	0.57
	recruited	0.0007263720809418	0.57
	spatial	6,39E+09	0.57
	episodic memory	0.0404192780857426	0.57

	types	0.0009317917298981	0.57
	cortex dlpfc	0.030293790388973	0.57
	actions	0.0048695164501389	0.57
	dlpfc	0.0157177046282561	0.57
	line	0.0256504367004478	0.57
	directed	0.0327706761470243	0.57
	encoding	0.0010430472912242	0.57
	trials	0.0009469194297731	0.57
	trial	0.0087184586424065	0.57
	involved	6,03E+07	0.57
	middle frontal	0.0054809210459356	0.57
	widespread	0.0460008750526656	0.57
	nature	0.0317838786063189	0.57
	episodic	0.0265543887768379	0.56
	active	0.0026101531491998	0.56
	number	0.002348741222503	0.56
	performance	2,89E+07	0.56
	reaction	0.0215385206553335	0.56
	items	0.0490043000568259	0.56
	simple	0.0351380340684272	0.56
	event	0.0001608827967414	0.56
	location	0.0464066199920029	0.56
	common	0.0040661671419808	0.56

	performing	0.0209670787928854	0.56
	frontal cortex	0.0177836001451104	0.55
	movements	0.0314714879392766	0.55
	components	0.0222533501316361	0.55
	better	0.0318331818597685	0.55
	stimulus	0.0007566575874642	0.55
	selective	0.0332609487191956	0.55
	experiment	0.0175839934856028	0.55
	language	0.0091445496089357	0.55
	perceptual	0.027742307944059	0.55
	recognition	0.0189528049580056	0.55
	lateral	0.0048272505288866	0.55
	information	0.000343506939444	0.55
	dorsal	0.0032081640051763	0.55
	presented	0.0042931724309428	0.55
	activations	0.0074727676596825	0.55
	inferior frontal	0.0032081640051763	0.55
	occipital	0.0258295603484509	0.54
	superior	0.0066097429894065	0.54
	motor	0.0145346975497053	0.54
	greater	0.0251460970892694	0.53
	networks	0.0427412235755313	0.53
	control	0.0141940103746489	0.53
RhIP1	calculation	1,75E+08	0.78

	parietal lobules	3,58E+09	0.76
	memory load	4,95E+09	0.76
	attention network	6,40E+06	0.74
	lateral parietal	0.0004747786057839	0.73
	subtraction	0.0001416319816999	0.73
	visuo spatial	0.0033021840295667	0.72
	intraparietal	4,76E-09	0.72
	navigation	0.0050042265546424	0.72
	sulci	0.0050042265546424	0.72
	intraparietal sulcus	1,59E-04	0.72
	fronto parietal	6,30E+00	0.72
	sulcus ips	3,34E+09	0.72
	symbolic	0.0089396162580459	0.71
	ips	2,14E+09	0.71
	numbers	0.0015707413679358	0.70
	frontal eye	9,62E+10	0.70
	arithmetic	0.0089396162580459	0.70
	response selection	0.0079922987045936	0.70
	paced	0.0089396162580459	0.69
	greater extent	0.0402196903449971	0.69
	prefrontal parietal	0.0006282771085954	0.69
	anterior intraparietal	0.023555150394602	0.69
	dorsal attention	0.0132594391492267	0.69

	ppc	0.0132594391492267	0.69
	parietal network	5,97E+10	0.69
	eye fields	0.0079922987045936	0.69
	native	0.0014379916527051	0.69
	working memory	2,02E-05	0.69
	cortex parietal	0.0299518082121871	0.69
	parietal	9,80E-26	0.69
	prefrontal cortices	4,93E+10	0.69
	lobules	0.0050042265546424	0.69
	working	1,56E-04	0.68
	familiarity	0.0004877204962255	0.68
	compensation	0.0388694578830417	0.68
	frontoparietal	3,27E+09	0.68
	separated	0.012496712398483	0.68
	maintain	0.0007961602505045	0.68
	posterior parietal	6,86E+06	0.68
	parietal cortex	4,71E-02	0.67
	risky	0.0468765010294632	0.67
	numerical	0.0299518082121871	0.67
	retrieved	0.0299518082121871	0.67
	networks involved	0.0299518082121871	0.67
	distance	0.0072711013684418	0.67
	recognition memory	0.0119521303060675	0.67
	spatial attention	0.0130131553465811	0.67

	maintained	0.0073492400951366	0.67
	shapes	0.0172572206177438	0.67
	frontal lobe	0.0024660942878638	0.67
	orienting	0.0177216467311389	0.66
	memory task	1,52E+11	0.66
	memory tasks	0.0419441998322117	0.66
	maintenance	5,00E+10	0.66
	task difficulty	0.019741229646689	0.66
	interact	0.0013821994143389	0.66
	attentional control	0.0363417955079779	0.66
	english	0.0256140540469364	0.66
	control processes	0.0279582070414212	0.66
	recollection	0.0279582070414212	0.66
	recruit	0.0133527429945194	0.65
	color	0.0102019651094894	0.65
	memory wm	0.0073492400951366	0.65
	depends	0.0045209046064594	0.64
	fronto	2,34E+08	0.64
	demands	1,52E+11	0.64
	identity	0.0419441998322117	0.64
	difficulty	0.0008739766144901	0.64
	forms	0.0187425143950204	0.64
	inferior parietal	3,04E+06	0.64

	ventrolateral prefrontal	0.0066439187213903	0.64
	encoded	0.0204270174796078	0.64
	attentional	2,53E+10	0.63
	memory retrieval	0.0431305279463301	0.63
	memory	2,77E+01	0.63
	visuospatial	0.0242059511444875	0.63
	parietal lobe	0.0050042265546424	0.63
	resources	0.013402760925537	0.63
	load	0.0081309227988449	0.63
	recruited	1,77E+10	0.63
	perspective	0.0461908413336598	0.63
	tasks	5,51E+05	0.62
	wm	0.0292803155039769	0.62
	sulcus	7,43E+08	0.61
	frontal parietal	0.0261914254730047	0.61
	parietal lobule	0.0016321733657424	0.61
	executive	0.0018237406916215	0.61
	dorsolateral	4,39E+10	0.61
	episodic	0.0242059511444875	0.61
	superior parietal	0.0089635098444327	0.60
	task	5,84E+03	0.60
	dorsolateral prefrontal	0.0011468881126034	0.60
	extent	0.0098509210785262	0.60
	pfc	0.0401224325020786	0.60

	spatial	0.0001560110338353	0.60
	lobule	0.0058938176281611	0.60
	given	0.0375088929672089	0.60
	retrieval	0.0050042265546424	0.60
	required	0.019741229646689	0.59
	attention	0.0007048007745831	0.58
	processes	0.0007888808462515	0.58
	prefrontal	9,32E+09	0.57
	prefrontal cortex	0.0007341867779616	0.57
	performance	0.0018237406916215	0.57
	contrast	0.0204270174796078	0.57
	frontal	0.0011671541097969	0.56
	network	0.0018237406916215	0.56
	networks	0.0375088929672089	0.56
	inferior	0.0088432573941153	0.56
	control	0.0319509387854329	0.55
RhIP2	calculation	2,47E+04	0.80
	anterior intraparietal	1,61E+08	0.77
	index finger	1,02E+10	0.76
	numerical	6,17E+07	0.75
	sulcus ips	6,67E+04	0.75
	numbers	1,07E+09	0.75
	ips	2,49E+02	0.74

	memory load	2,20E+11	0.74
	symbolic	0.0002288933156268	0.74
	intraparietal sulcus	4,43E-15	0.74
	intraparietal	1,31E-15	0.73
	superior inferior	0.0006295022396894	0.73
	pointing	0.0010898261422428	0.73
	greater extent	0.0017751189832007	0.72
	cortex ppc	0.0003967518414118	0.72
	arithmetic	0.000486156614574	0.72
	prefrontal parietal	4,05E+09	0.71
	mental imagery	0.0027850361254434	0.71
	ppc	0.0008631237278991	0.71
	parietal cortex	5,55E-17	0.71
	ventral premotor	9,73E+08	0.71
	distance	7,61E+09	0.71
	parietal	1,28E-42	0.71
	memory wm	6,25E+06	0.71
	eye fields	0.0005871717489511	0.71
	held	0.0029805811461124	0.70
	rehearsal	0.0221827762528806	0.70
	grasping	0.0082879794233338	0.69
	inferior parietal	2,21E-11	0.69
	fronto parietal	3,04E+05	0.69
	shapes	0.0008773731487681	0.69

	cortex frontal	0.0337256077553238	0.69
	cortical network	0.0005695369011005	0.69
	parietal lobules	0.0273524989175073	0.69
	finger movements	0.0273524989175073	0.69
	parietal frontal	0.0013065438874205	0.68
	attend	0.0313515679279431	0.68
	task task	0.0114166846269008	0.68
	young healthy	0.045818056321699	0.68
	cortical networks	0.0132063357867898	0.68
	tactile	6,90E+10	0.68
	working memory	8,39E-05	0.68
	wm	5,33E+08	0.68
	paced	0.0216426608650865	0.68
	working	7,76E-03	0.67
	task difficulty	0.0034411003643362	0.67
	finger	3,55E+10	0.67
	spatial attention	0.0056349227567305	0.67
	rt	0.0296028345042084	0.67
	attention network	0.0030700109030491	0.67
	joint	0.0329606807062616	0.67
	parietal network	0.000882892042442	0.67
	posterior parietal	6,17E+07	0.67
	imagery	2,04E+10	0.67

	absent	0.0155426132716146	0.67
	updating	0.0296028345042084	0.67
	conjunction	7,17E+09	0.66
	load	1,33E+11	0.66
	frontoparietal	1,73E+11	0.66
	imagined	0.0211550418637894	0.66
	subserving	0.0280974586025606	0.66
	parieto	0.0072464375959615	0.65
	virtual	0.0200803789242235	0.65
	coding	0.0021972641861698	0.65
	operations	0.0152531244424571	0.65
	parietal cortices	0.0048700152225782	0.65
	sulcus	1,54E+03	0.64
	moving	0.0156454102878401	0.64
	sequential	0.0412767680934319	0.64
	resources	0.0010819461870082	0.64
	attentional	7,01E+07	0.64
	irrelevant	0.0092605672146813	0.64
	superior parietal	4,05E+09	0.64
	execution	0.0004992452592288	0.64
	position	0.0250486154546345	0.64
	fields	0.0352830604385497	0.64
	task performance	9,55E+10	0.64
	prefrontal cortices	0.0201178359780101	0.64

	maintenance	0.0018614112052283	0.64
	task functional	0.0237397001041796	0.63
	overlapping	0.0010152327916202	0.63
	fronto	9,73E+08	0.63
	parietal lobule	4,05E+09	0.63
	frontal parietal	0.0008076720644184	0.63
	tasks	1,53E+03	0.63
	parietal lobe	0.0036462897017553	0.63
	discrimination	0.0052755579513774	0.63
	manipulation	0.0106811322592953	0.63
	engage	0.0144015496050895	0.63
	modality	0.004437359801131	0.62
	critically	0.0391564778339209	0.62
	number	3,55E+10	0.62
	premotor	9,73E+08	0.62
	interact	0.0414003987619871	0.62
	visuospatial	0.041257643414375	0.62
	spatial	3,95E+08	0.62
	difficulty	0.0097249377250062	0.62
	lobule	9,55E+10	0.61
	parametric	0.0200803789242235	0.61
	make	0.0459640337745447	0.61
	required	0.0006298122372975	0.61

	target	0.0001821186195397	0.61
	simple	0.0072464375959615	0.61
	overlap	0.0220471983405019	0.61
	requires	0.0227791849107121	0.61
	task	5,69E+01	0.61
	demands	0.0043817584975615	0.61
	attention	5,71E+08	0.60
	dorsolateral prefrontal	0.0002978827128356	0.60
	dorsolateral	0.000252213577936	0.59
	reaction	0.0329606807062616	0.59
	movements	0.0180489373681776	0.59
	hand	0.005997452345653	0.59
	inferior	2,17E+08	0.59
	engaged	0.0156454102878401	0.59
	networks	0.0001734336414606	0.58
	general	0.0180620725785526	0.58
	supplementary	0.0371348330228191	0.58
	recruited	0.0480158958368817	0.58
	processes	0.0003386510462036	0.57
	memory	0.0002024508308021	0.57
	experiment	0.0492663107870799	0.57
	motor	0.0003226997816394	0.57
	network	2,18E+11	0.57
	common	0.0288375094597738	0.57

	cortices	0.0220471983405019	0.57
	activations	0.0386675277944673	0.56
	conditions	0.0313515679279431	0.56
	stimulus	0.0480158958368817	0.56
	frontal	0.0014374617576017	0.56
	visual	0.0220471983405019	0.55
	prefrontal	0.0222511922890872	0.55
RhIP3	sulcus ips	1,26E-05	0.75
	ips	1,97E-09	0.74
	intraparietal	2,79E-50	0.74
	calculation	6,21E+07	0.74
	intraparietal sulcus	5,84E-41	0.74
	arithmetic	2,22E+06	0.73
	symbolic	4,01E+08	0.73
	eye fields	3,29E+04	0.72
	parietal lobules	2,11E+08	0.72
	rotation	5,53E+07	0.72
	numerical	1,29E+07	0.72
	memory load	3,30E+08	0.72
	subtraction	3,39E+08	0.71
	numbers	1,29E+08	0.71
	superior inferior	1,14E+11	0.71
	visuo spatial	2,36E+10	0.71

	dorsal attention	1,03E+10	0.70
	ppc	1,03E+10	0.70
	frontal eye	1,49E+05	0.70
	parietal network	1,37E+04	0.70
	parietal frontal	4,95E+06	0.70
	upcoming	6,86E+09	0.70
	preparation	4,28E+05	0.70
	prefrontal parietal	3,05E+05	0.70
	eye movements	1,11E+10	0.69
	parietal	5,45E-76	0.69
	cortex ppc	1,79E+11	0.69
	eye field	1,79E+11	0.69
	cortex parietal	3,94E+10	0.69
	fronto parietal	2,95E-05	0.69
	preparatory	0.0001118570684247	0.69
	saccade	0.0003580070906406	0.69
	spatial information	0.0003580070906406	0.69
	lobules	2,84E+09	0.68
	anterior intraparietal	0.0002921950182235	0.68
	task difficulty	6,45E+08	0.68
	motor imagery	7,21E+10	0.68
	visual attention	1,21E+11	0.68
	posterior parietal	2,21E-03	0.68
	storage	0.0001085623724946	0.68

	attention network	3,07E+09	0.68
	allocation	0.000344221996445	0.68
	load	1,23E+02	0.68
	visuospatial	2,46E+06	0.67
	dot	0.0021134982306878	0.67
	navigation	0.0021134982306878	0.67
	mental imagery	0.0013273181578947	0.67
	task irrelevant	4,97E+09	0.67
	current functional	0.0019085433482274	0.67
	visuo	9,48E+09	0.67
	verbal working	0.0006249768216971	0.67
	demand	8,99E+07	0.67
	shifting	0.0001391236398291	0.67
	frontoparietal	3,02E+04	0.67
	cortical involved	0.0056132783849612	0.67
	memory wm	8,32E+06	0.67
	paced	0.0006884103482466	0.67
	superior parietal	2,65E-03	0.67
	saccades	0.0068894267177536	0.66
	pointing	0.0068894267177536	0.66
	parietal lobes	0.0022364127419396	0.66
	parietal cortex	3,64E-14	0.66
	frontoparietal network	0.00044992254397	0.66

	irrelevant	9,02E+07	0.66
	spatial attention	6,18E+10	0.66
	eye	3,05E+03	0.66
	imagery	3,98E+06	0.66
	fields	1,14E+11	0.66
	wm task	0.0036167166982407	0.66
	attentional control	0.0002820181846919	0.66
	span	0.010939582626984	0.65
	parietal cortices	1,10E+10	0.65
	cortical network	0.0002175517339082	0.65
	working memory	4,01E-09	0.65
	hands	0.001498702122448	0.65
	orienting	0.0003017165535245	0.65
	flexibly	0.020757456331239	0.65
	rehearsal	0.020757456331239	0.65
	motor pre	0.0245276812926163	0.65
	greater extent	0.0245276812926163	0.65
	watched	0.0101915290848818	0.65
	working	1,29E-06	0.65
	finger movements	0.01840989676638	0.65
	held	0.006458978489146	0.65
	serial	0.0021261863235384	0.65
	visuomotor	0.0008486267862191	0.64
	response selection	0.008856065314453	0.64

	kinds	0.0186888773842824	0.64
	updating	0.0056467250174376	0.64
	manipulation	7,27E+08	0.64
	resources	3,60E+08	0.64
	color	2,32E+11	0.64
	letters	0.0025808022538281	0.64
	material	0.0003914288492619	0.64
	sulci	0.0334813316411622	0.64
	internally	0.0077471446653095	0.64
	response times	0.0250940071249602	0.64
	spl	0.0250940071249602	0.64
	execution	5,39E+08	0.64
	grasping	0.0250940071249602	0.64
	ventral premotor	0.000542776035116	0.64
	younger adults	0.0162151090033222	0.64
	action observation	0.0090132605991075	0.64
	attentional	1,34E+02	0.64
	operation	0.0444166805800021	0.64
	away	0.0290041351078716	0.64
	reaching	0.0220914075074218	0.64
	readers	0.0118684321969944	0.64
	flexible	0.0029791655207968	0.64
	moving	0.0005324949846116	0.63

	premotor	1,56E+00	0.63
	fixation	0.004098767976016	0.63
	subserved	0.0441951199009819	0.63
	distractor	0.0441951199009819	0.63
	task relevant	0.0015859780538257	0.63
	visually	5,57E+09	0.63
	attention task	0.0380076023318907	0.63
	instructions	0.0245276812926163	0.63
	guided	0.0007971875019683	0.63
	whilst	0.0330531982671756	0.63
	sulcus	1,61E-02	0.63
	wm	1,29E+10	0.63
	dorsal premotor	0.0017284630193518	0.63
	tracking	0.0084311334075519	0.63
	term memory	0.0016525614148841	0.63
	network involved	0.0036974469550737	0.63
	operations	0.0043196257930521	0.63
	vision	0.014929615215773	0.62
	selective attention	0.0122921289021894	0.62
	maintenance	2,82E+11	0.62
	connectivity analyses	0.0056386549555099	0.62
	just	0.0353100603421102	0.62
	mirror	0.0008928930305179	0.62
	distance	0.0103535246065337	0.62

	exact	0.0394715116068045	0.62
	neuron	0.0334813316411622	0.62
	ipl	0.0110854963091484	0.62
	simply	0.0384000545213305	0.62
	parametrically	0.0441951199009819	0.62
	difficulty	1,79E+11	0.62
	locations	0.0012313127275134	0.62
	endogenous	0.0499661201557974	0.62
	discrimination task	0.0499661201557974	0.62
	tasks	4,10E-06	0.62
	coordination	0.0201107904344552	0.62
	reaction time	0.000466454130898	0.62
	digit	0.0488559444406682	0.62
	short term	0.0070249197016864	0.62
	conclude	1,22E+11	0.62
	spatial	1,23E+02	0.62
	younger	0.0057904826808285	0.62
	maintained	0.0155401761074333	0.61
	forms	0.002691250544066	0.61
	integrate	0.0249376021513741	0.61
	stroop task	0.033776809721749	0.61
	maintain	0.0105997653714836	0.61
	parieto	0.0105997653714836	0.61

	premotor cortex	9,02E+08	0.61
	cognitive tasks	0.0201558295287203	0.61
	demands	1,03E+10	0.61
	extrastriate	0.0033676126735981	0.61
	attended	0.0320855467807823	0.61
	lateralized	0.0003470176655869	0.61
	manipulating	0.0431822423307712	0.61
	parietal lobe	0.0002600587573393	0.61
	task	2,37E-10	0.61
	orthographic	0.0488559444406682	0.61
	oriented	0.0488559444406682	0.61
	frontal parietal	7,84E+10	0.61
	lateral occipital	0.0071663765713366	0.61
	interference	0.0008213073915628	0.61
	fronto	1,36E+08	0.61
	shapes	0.0496701286144517	0.61
	observation	0.0008304078190042	0.61
	faster	0.0063861382751105	0.61
	stroop	0.0110738819586143	0.61
	goal directed	0.0133232893314261	0.61
	demanding	0.0272671612691243	0.61
	movements	2,09E+09	0.61
	reaction	3,04E+10	0.60
	skill	0.0499661201557974	0.60

	attention	9,00E+00	0.60
	efficient	0.0115873989686949	0.60
	capacity	0.0045598996993716	0.60
	task demands	0.0444166805800021	0.60
	familiarity	0.0342487912401723	0.60
	dorsal ventral	0.0464122837309073	0.60
	middle occipital	0.0499661201557974	0.60
	direction	0.0072078286642904	0.60
	shape	0.0152494152742122	0.60
	reaction times	0.0298586598828874	0.60
	visual	2,06E+00	0.60
	lateral prefrontal	0.004428989723145	0.60
	cognitive processes	0.0106931261248403	0.59
	inferior parietal	6,93E+07	0.59
	occipito	0.041976064966869	0.59
	shift	0.0177454714632405	0.59
	requiring	0.0213115565308736	0.59
	version	0.0411023835532962	0.59
	effort	0.0499661201557974	0.59
	task performance	0.0012003710034119	0.59
	object	1,14E+11	0.59
	recruitment	0.0003966856714007	0.59
	cue	0.0021261863235384	0.59

	rely	0.0245276812926163	0.59
	executive control	0.0433067145930793	0.59
	item	0.0272106513982622	0.59
	engaged	3,70E+10	0.59
	contrasted	0.0355336471026326	0.59
	hand	1,70E+11	0.59
	executive	6,36E+10	0.59
	objects	0.0002239989701907	0.59
	perform	0.0165927329950263	0.59
	performing	0.0002302009127976	0.59
	directed	0.0109700485583553	0.58
	required	0.0005988124552302	0.58
	times	0.0113374327211944	0.58
	targets	0.0265410476153781	0.58
	cognitive control	0.0024475784242152	0.58
	generation	0.0333043880055162	0.58
	older adults	0.0424192754372868	0.58
	target	0.0002838205910702	0.58
	conjunction	0.0468611658544044	0.58
	parietal lobule	0.0010015397118457	0.58
	occipital cortex	0.0354411920185757	0.58
	network	7,93E+02	0.58
	activations	3,48E+10	0.58
	performance	3,11E+07	0.58

	ventrolateral	0.0155618515474452	0.58
	lobule	0.0007730531024682	0.58
	reading	0.0105997653714836	0.58
	mental	0.0008210888010404	0.58
	supporting	0.0093042510650692	0.57
	memory	1,19E+08	0.57
	recruited	0.0014970138007296	0.57
	perceptual	0.000991383598273	0.57
	number	0.001261638178349	0.57
	goal	0.0115195095234492	0.57
	action	0.001498702122448	0.57
	representation	0.0026972109655885	0.57
	representations	0.0026850608157959	0.57
	dorsal	2,95E+10	0.57
	additional	0.0062186218071705	0.57
	dorsolateral	0.000410535641664	0.57
	occipital	0.0001620367105668	0.57
	verbal	0.0134257053131597	0.57
	processes	9,97E+08	0.56
	directly	0.0464003921765433	0.56
	dorsolateral prefrontal	0.0049330999255304	0.56
	involving	0.0325762986353066	0.56
	stimulus	0.0003640093415506	0.56

	extent	0.0256679509859749	0.56
	supplementary	0.0181826918808259	0.56
	involved	4,81E+09	0.56
	common	0.0066587190647854	0.56
	involvement	0.0262300472695614	0.56
	motor	0.0002022928770292	0.55
	control	3,27E+10	0.55
	cerebral	0.0421285055446073	0.55
	contrast	0.003783584152749	0.55
	information	0.001498702122448	0.55
	cognitive	0.0005519002929914	0.55
	frontal	0.0001496690223219	0.55
	conditions	0.0137735678820013	0.55
	correlates	0.0225746904852798	0.54
	lateral	0.0260598278642695	0.54
	networks	0.0153658665240022	0.54
	presented	0.0334813316411622	0.54
	inferior	0.0062658742235989	0.54
	prefrontal	0.0494898467704159	0.53

Supporting Information 3: Table of terms of the systems-level decoding for each seed region and number of cortical regions that were associated with the terms in conjunction with the given seed region.

Seed Region	Terms	Number of Regions
LhIP1	anterior intraparietal	57
	arithmetic	72
	attention network	44
	behavioral evidence	19
	bilinguals	1
	calculation	68
	characters	14
	concrete	2
	control processes	4
	cortex ppc	58
	cortical networks	5
	domain general	8
	dyslexia	5
	exact	1
	expertise	1
	eye field	24
	flexibility	1
	flexibly	53
	frontal eye	3
	fronto parietal	13
	frontoparietal	79
	frontoparietal network	70

	grasping	1
	implementation	3
	inferior parietal	3
	inferior superior	15
	intraparietal	76
	intraparietal sulcus	77
	ips	70
	lateral parietal	1
	list	1
	load	2
	loop	2
	memory load	70
	memory processes	1
	memory retrieval	8
	memory wm	51
	motor pre	6
	native	18
	negative feedback	16
	networks involved	3
	numbers	81
	numerical	62
	operations	2
	opercular	5
	parietal	50

	parietal cortex	4
	parietal frontal	4
	parietal lobules	17
	parietal network	68
	phonological	14
	portions	1
	posterior parietal	53
	ppc	59
	pre supplementary	24
	prefrontal parietal	39
	preparatory	68
	rehearsal	3
	relational	1
	reliance	59
	requirements	2
	response times	18
	retrieved	1
	rule	1
	rules	5
	shifting	39
	speakers	1
	subtraction	78
	sulcus ips	78

	switch	6
	switching	17
	symbolic	84
	target detection	11
	upcoming	2
	ventral premotor	5
	verbal working	4
	visual motion	1
	visuo spatial	25
	wm task	1
	working memory	21
LhIP2	acts	4
	anterior intraparietal	82
	arithmetic	67
	calculation	61
	characters	5
	chinese	1
	conflicting	5
	cortex parietal	19
	cortex ppc	25
	current functional	4
	deactivated	2
	demands	5
	distractor	1

	distributed network	2
	domain general	2
	expectancy	21
	frontal cortices	1
	frontal lobes	5
	fronto parietal	5
	frontoparietal	2
	frontoparietal network	28
	gestures	13
	grasping	31
	greater extent	3
	hands	17
	imagine	1
	imitation	7
	implementation	63
	inferior parietal	1
	intraparietal	57
	intraparietal sulcus	54
	involved cognitive	1
	ips	5
	judge	5
	lateral frontal	5
	list	9

	lobules	56
	memory load	3
	memory processes	1
	memory tasks	1
	memory wm	12
	mental imagery	14
	mirror neuron	3
	motor imagery	6
	motor pre	2
	native	4
	negative feedback	10
	networks involved	11
	neuron	2
	nouns	1
	noxious	3
	numbers	74
	operations	2
	paced	1
	parietal	39
	parietal frontal	16
	parietal lobes	3
	parietal lobules	81
	parietal network	11
	parietal temporal	3

	parieto occipital	1
	phonological	2
	posterior parietal	10
	ppc	41
	pre sma	12
	pre supplementary	2
	prefrontal parietal	72
	preparatory	2
	producing	2
	rehearsal	72
	relational	9
	reliance	1
	relied	28
	remember	2
	requirements	65
	response time	4
	response times	10
	rotation	1
	rules	4
	semantics	8
	stimulus response	12
	strategic	11
	striking	6

	subtraction	74
	successive	70
	sulcus ips	10
	superior inferior	5
	switch	2
	switching	1
	symbolic	77
	tactile	4
	task irrelevant	4
	updating	1
	valid	1
	ventral premotor	56
	verbal working	12
	verbs	1
	visual word	1
	visually presented	1
	visuo spatial	11
	vlpfc	1
	working memory	2
LhIP3	action observation	1
	allocation	3
	anterior intraparietal	93
	arithmetic	112
	attention network	50

	bilinguals	4
	calculation	103
	characters	14
	computation	3
	conflicting	61
	coordination	1
	cortex parietal	57
	cortex ppc	114
	domain general	2
	english	7
	execution	12
	expectancy	2
	eye field	1
	eye fields	38
	flexibly	22
	frontal eye	2
	fronto parietal	101
	frontoparietal	10
	frontoparietal network	108
	grasping	68
	inferior superior	57
	intraparietal	112
	intraparietal sulcus	113

	ips	110
	letters	2
	lobules	2
	memory load	97
	memory tasks	1
	memory wm	5
	native	4
	navigation	1
	numbers	113
	numerical	117
	orienting	2
	orthographic	3
	parietal	95
	parietal cortex	1
	parietal frontal	107
	parietal lobules	111
	parietal network	114
	posterior parietal	32
	ppc	114
	prefrontal parietal	7
	preparatory	112
	readers	8
	recognition memory	1
	rehearsal	4

	relied	4
	remember	1
	response times	61
	rotation	12
	rules	1
	saccades	6
	shifting	71
	spatial attention	3
	spl	1
	stimulus driven	7
	storage	11
	stored	1
	sulcus ips	111
	superior inferior	74
	superior parietal	41
	switch	1
	symbolic	118
	task irrelevant	1
	task task	19
	ventral premotor	1
	verbal working	65
	visual attention	2
	visual cortices	1

	visual word	5
	visually presented	1
	visuo	21
	visuo spatial	52
	wm task	6
RhIP1	absent	2
	allocation	1
	anterior intraparietal	8
	arithmetic	29
	attention network	67
	attention task	4
	behavioral evidence	5
	calculation	59
	computation	1
	concrete	2
	control conditions	6
	control processes	2
	cortex ba	1
	cortex parietal	13
	cortex ppc	5
	current functional	2
	differing	4
	distractor	1
	domain general	2

	dorsal attention	23
	dorsolateral pfc	5
	english	4
	environments	1
	executive functions	1
	eye field	7
	eye fields	9
	familiarity	12
	flexibly	1
	formed	1
	frontal eye	27
	frontal lobes	11
	fronto parietal	69
	frontoparietal	20
	frontoparietal network	8
	greater extent	27
	gyrus medial	1
	implementation	4
	instruction	1
	intraparietal	53
	intraparietal sulcus	46
	ips	42
	judgment task	2

	lateral medial	6
	lateral parietal	58
	linguistic	1
	lobules	23
	maintain	19
	maintaining	1
	maintenance	1
	matrix	3
	memory load	67
	memory processes	4
	memory tasks	9
	memory test	1
	memory wm	2
	motor pre	1
	motor response	1
	native	18
	navigation	41
	negative feedback	1
	networks involved	13
	neurofunctional	2
	numbers	51
	numerical	13
	options	1
	orienting	3

	paced	20
	parietal	17
	parietal frontal	1
	parietal lobules	70
	parietal network	33
	pitch	1
	pointing	15
	portions	1
	posterior inferior	4
	posterior parietal	12
	ppc	19
	pre supplementary	1
	prefrontal cortices	11
	prefrontal parietal	34
	preparatory	4
	readers	1
	recognition memory	8
	recollection	4
	recruit	2
	rehearsal	2
	relational	10
	remember	10
	requirements	4

	response selection	19
	retrieved	10
	reversal	8
	risky	8
	rotation	1
	saccade	1
	separated	12
	shifted	3
	shifting	1
	solving	3
	spatial attention	7
	spl	1
	stimulus driven	2
	subtraction	63
	sulci	33
	sulcus ips	49
	superior inferior	3
	symbolic	40
	task difficulty	7
	task required	1
	task task	1
	took	1
	unlike	3
	ventrolateral prefrontal	1

	verbal working	1
	visuo spatial	54
	working memory	18
	younger adults	3
RhIP2	allocation	11
	anterior intraparietal	17
	arithmetic	91
	attention network	6
	calculation	80
	cortex parietal	37
	cortex ppc	44
	cortical involved	1
	demand	3
	dorsal attention	78
	dot	6
	eye field	49
	eye fields	85
	eye movements	39
	flexibly	1
	frontal eye	78
	fronto parietal	49
	frontoparietal	3
	frontoparietal network	5

	grasping	1
	greater extent	3
	intraparietal	90
	intraparietal sulcus	91
	ips	92
	load	3
	lobules	24
	memory load	89
	memory wm	1
	mental imagery	15
	motor imagery	9
	motor pre	2
	navigation	5
	numbers	86
	numerical	96
	paced	3
	parietal	56
	parietal frontal	82
	parietal lobes	1
	parietal lobules	91
	parietal network	88
	pointing	5
	posterior parietal	8
	ppc	71

	prefrontal parietal	67
	preparation	80
	preparatory	32
	rehearsal	1
	response times	3
	rotation	92
	saccade	15
	saccades	1
	shifting	3
	single subject	1
	spatial information	20
	storage	10
	subtraction	83
	sulcus ips	91
	superior inferior	88
	symbolic	95
	task difficulty	22
	task irrelevant	6
	upcoming	72
	verbal working	3
	visual attention	11
	visuo	1
	visuo spatial	80

	watched	2
	wm task	1
RhIP3	absent	4
	actually	3
	anterior intraparietal	129
	arithmetic	77
	attend	27
	attention network	5
	calculation	119
	convergence	1
	coordination	1
	correspond	1
	cortex dorsolateral	1
	cortex lateral	10
	cortex ppc	96
	cortical involved	6
	cortical networks	12
	cross modal	2
	current functional	1
	deactivated	2
	distractor	1
	dot	3
	effortful	1
	eye fields	54

	fewer	1
	fields	1
	finger movements	16
	force	5
	fronto parietal	54
	frontoparietal	3
	grasping	42
	greater extent	98
	hands	2
	high functioning	3
	imagery	4
	imagine	1
	imagined	5
	index finger	121
	instruction	2
	insula anterior	1
	intraparietal	117
	intraparietal sulcus	124
	ips	123
	joint	7
	lateral parietal	1
	list	1
	load	3

	lobule ipl	6
	memory load	120
	memory wm	77
	mental imagery	90
	motor imagery	6
	moving	2
	navigation	10
	negative feedback	1
	nogo	1
	numbers	126
	numerical	130
	observers	1
	oddball	1
	operation	3
	operations	1
	paced	19
	parietal	85
	parietal frontal	26
	parietal lobules	35
	parietal network	9
	pointing	104
	posterior parietal	3
	ppc	79
	prefrontal parietal	88

	producing	1
	reaching	1
	rehearsal	24
	repetition suppression	1
	replicate	2
	requirements	10
	response selection	1
	response times	10
	reversal	2
	rotation	2
	s1	2
	saccades	1
	segregated	5
	shapes	25
	single subject	1
	somatosensory cortices	1
	spatial attention	10
	spl	7
	stimulus response	2
	subtraction	8
	sulci	3
	sulcus ips	126
	superior inferior	115

	symbolic	124
	tactile	11
	target detection	13
	task difficulty	12
	task required	1
	task task	10
	temporal frontal	5
	temporal inferior	8
	transformation	3
	updating	2
	ventral premotor	84
	visual cortices	7
	visual field	1
	visual stimulus	1
	visuo spatial	1
	watched	3
	wm	3
	wm task	7
	working memory	7

Supporting Information 4: All Neurosynth topics with at least one term grouped into that topic. Please note, terms denoting anatomical terms were excluded. Topics described in the main section of the article as cognitive systems associated with the IPS are highlighted in green.

Topics	LhIP1	LhIP2	LhIP3	RhIP1	RhIP2	RhIP3
Topic 2	loop					
Topic 4	target detection	response time; stimulus response		instruction		instruction; repetition suppression; stimulus response; target detection; visual stimulus
Topic 6	list	list		pitch		list
Topic 7	negative feedback	negative feedback; stimulus response		negative feedback		force; negative feedback; stimulus response
Topic8				recruit		
Topic 9	load; memory load; memory wm; verbal working; wm task; working memory	memory load; memory wm; verbal working; working memory	memory load; memory wm; storage; verbal working; wm task	executive functions; maintain & maintenance; memory load; memory wm; verbal working; working memory	load; memory load; memory wm; storage; verbal working; wm task	load; memory load; memory wm; wm; wm task; working memory
Topic 11	expertise					
Topic 13		stimulus response				stimulus response
Topic14				memory test		
Topic 15	response times	demands; response time & response times	response times	attention task; recruit; task difficulty	demand; response times; task difficulty	response times; task difficulty
Topic 16	preparatory	preparatory; response time; stimulus response	execution; preparatory	motor response; preparatory; response selection	preparation; preparatory	nogo; response selection; stimulus response
Topic 17		motor imagery	execution; visuo		motor imagery; visuo	finger movements; force; imagery; motor imagery
Topic 18	arithmetic; calculation; numbers;	arithmetic; calculation; numbers;	arithmetic; calculation; numbers;	arithmetic; calculation; numbers;	arithmetic; calculation; numbers;	arithmetic; calculation; numbers;

	numerical; operations; subtraction; symbolic	operations; subtraction; symbolic	numerical; symbolic	numerical; subtraction; symbolic	numerical; subtraction; symbolic	numerical; operation; operations; subtraction; symbolic
Topic 19	grasping	gestures; grasping; imitation	action observation; execution; grasping		grasping	grasping
Topic 20	flexibility; switch; switching		switch	attention task		stimulus response
Topic 21		acts				wm
Topic 24				recruit		
Topic 25		rotation	rotation	rotation	rotation	rotation
Topic 28						operation
Topic 30				risky		
Topic 32		noxious; stimulus response; tactile				stimulus response; tactile
Topic 33	memory retrieval	remember	recognition memory; remember	familiarity; memory test; recognition memory; recollection; remember		
Topic 34		response time				
Topic 37	bilinguals; characters; dyslexia; native; phonological	characters; chinese; native; phonological; verbs; visual word	bilinguals; characters; english; native; orthographic; readers; visual word	english; linguistic; native; readers		
Topic 38	concrete	nouns; stimulus response				
Topic 40	visual motion			concrete		stimulus response
Topic 41		imagine; mental imagery	navigation	navigation	mental imagery; navigation	imagery;<br><br>imagine; imagined; mental imagery; navigation

Topic 42		tactile	letters			cross modal; tactile; visual stimulus
Topic 44			saccades	saccade	eye movements; saccade & saccades	saccades
Topic 45	visual motion	stimulus response				moving; shapes; stimulus response; visual stimulus
Topic 47	target detection	distractor; stimulus response	orienting; spatial attention; visual attention	attention task; distractor; orienting; response selection; spatial attention	visual attention	attend; distractor; response selection; spatial attention; stimulus response; target detection; visual stimulus
Topic 48	rule; rules; shifting	demands; rules	rules; shifting	executive functions; shifting	demand; shifting	

Supporting Information 5: Heatmaps displaying the percentage of overlaps between the topic surface maps and the 7Networks (and vice versa).

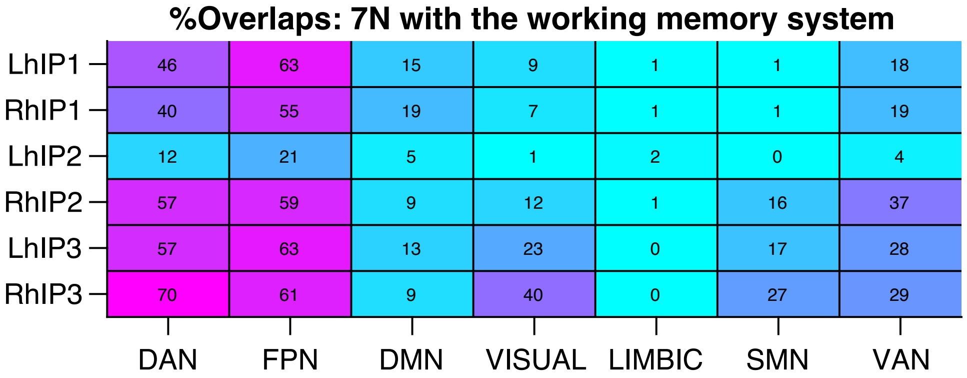

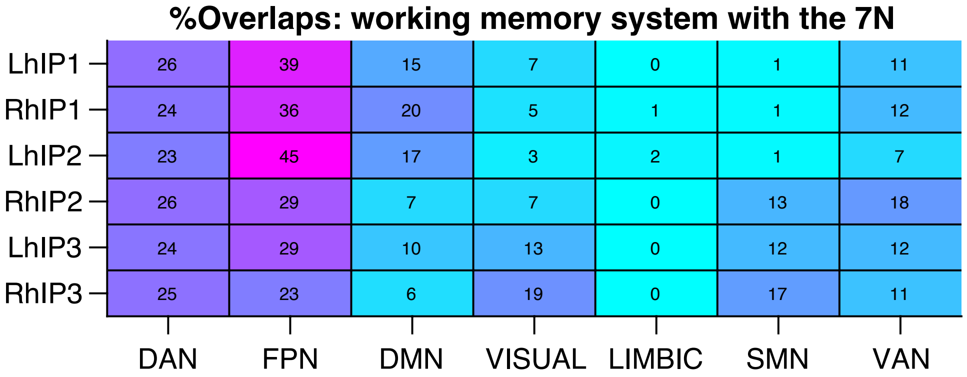

%Overlaps: 7N with the numeric cognition System

	DAN	FPN	DMN	VISUAL	LIMBIC	SMN	VAN
LhIP1	69	71	17	10	1	2	25
RhIP1	47	65	21	7	1	1	22
LhIP2	64	69	16	9	2	3	25
RhIP2	71	69	9	12	1	17	39
LhIP3	78	68	17	26	0	25	30
RhIP3	83	71	11	48	0	42	39

%Overlaps: numeric cognition system with the 7N

	DAN	FPN	DMN	VISUAL	LIMBIC	SMN	VAN
LhIP1	31	35	14	6	0	2	12
RhIP1	25	37	20	5	0	1	12
LhIP2	30	35	13	6	1	2	13
RhIP2	28	30	6	6	0	12	17
LhIP3	27	25	10	12	0	15	11
RhIP3	23	21	6	17	0	21	12

%Overlaps: 7Networks with the attention system

	DAN	FPN	DMN	VISUAL	LIMBIC	SMN	VAN
LhIP1	1	18	4	1	0	0	7
RhIP1	13	28	8	3	1	1	6
LhIP2	5	13	2	1	0	0	2
RhIP2	12	5	0	1	0	5	3
LhIP3	9	2	0	2	0	0	0
RhIP3	27	18	3	28	0	21	6

%Overlaps: attention systems with the 7N

	DAN	FPN	DMN	VISUAL	LIMBIC	SMN	VAN
LhIP1	3	54	21	2	0	0	20
RhIP1	19	44	21	5	0	1	9
LhIP2	19	51	15	6	0	0	9
RhIP2	39	19	2	3	0	29	9
LhIP3	66	14	0	20	0	0	0
RhIP3	21	15	4	28	0	28	5

%Overlaps: 7N with the grasping system

	DAN	FPN	DMN	VISUAL	LIMBIC	SMN	VAN
LhIP1	1	1	0	0	0	0	3
LhIP2	29	18	4	7	0	1	13
RhIP2	3	0	0	0	0	0	0
LhIP3	48	15	4	20	0	22	22
RhIP3	24	13	2	19	0	8	10

%Overlaps: grasping system with the 7N

	DAN	FPN	DMN	VISUAL	LIMBIC	SMN	VAN
LhIP1	16	20	0	0	0	0	63
LhIP2	36	24	9	12	0	3	17
RhIP2	80	1	0	18	0	0	0
LhIP3	30	10	5	16	0	25	15
RhIP3	26	15	4	27	0	16	11

%Overlaps: 7N with the language system

	DAN	FPN	DMN	VISUAL	LIMBIC	SMN	VAN
LhIP1	22	31	8	2	1	1	10
RhIP1	7	15	7	1	0	0	7
LhIP2	5	8	5	1	0	0	5
LhIP3	12	23	5	4	0	1	10

%Overlaps: language system with the 7N

	DAN	FPN	DMN	VISUAL	LIMBIC	SMN	VAN
LhIP1	26	39	17	3	0	2	13
RhIP1	15	36	28	4	1	1	16
LhIP2	18	31	29	3	0	2	17
LhIP3	19	39	15	7	0	2	17

%Overlaps: 7N with the recognition memory system

	DAN	FPN	DMN	VISUAL	LIMBIC	SMN	VAN
LhIP1	0	11	3	0	0	0	1
RhIP1	7	26	9	1	0	0	6
LhIP2	0	3	1	1	0	0	1
LhIP3	0	5	0	0	0	0	1

%Overlaps: recognition memory system with the 7N

	DAN	FPN	DMN	VISUAL	LIMBIC	SMN	VAN
LhIP1	2	64	30	0	0	0	3
RhIP1	11	47	28	3	0	0	11
LhIP2	4	46	20	12	0	0	18
LhIP3	0	75	5	0	0	0	21

%Overlaps: 7N with the nagivation system

	DAN	FPN	DMN	VISUAL	LIMBIC	SMN	VAN
RhIP1	16	39	13	4	1	0	8
LhIP2	4	16	7	1	0	1	9
RhIP2	11	19	1	2	0	0	8
LhIP3	4	1	0	0	0	1	0
RhIP3	59	46	8	38	0	21	31

%Overlaps: navigation system with the 7N

	DAN	FPN	DMN	VISUAL	LIMBIC	SMN	VAN
RhIP1	17	44	24	5	1	0	8
LhIP2	9	38	27	2	0	3	22
RhIP2	23	44	6	7	0	1	19
LhIP3	72	11	0	0	0	17	0
RhIP3	24	21	6	20	0	15	14

%Overlaps: 7N with the movements/shapes system

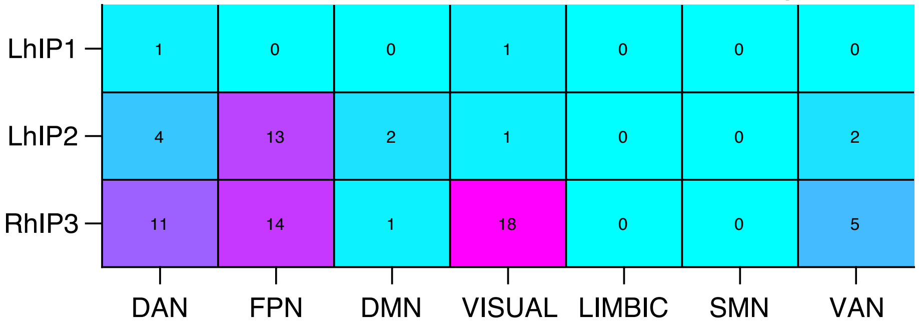

%Overlaps: movements/shapes system with the 7N

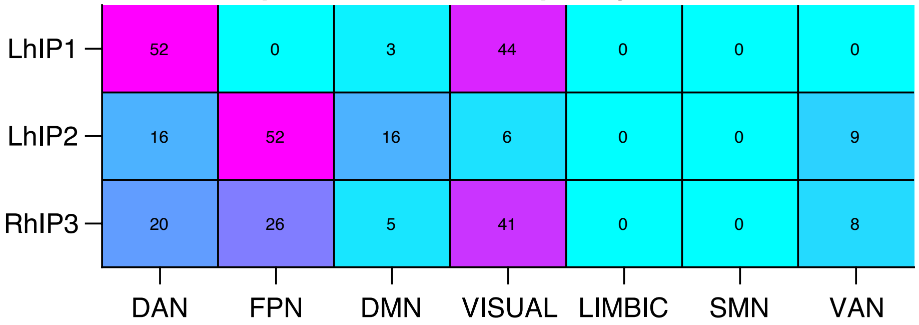

%Overlaps: 7N with the mental rotation system

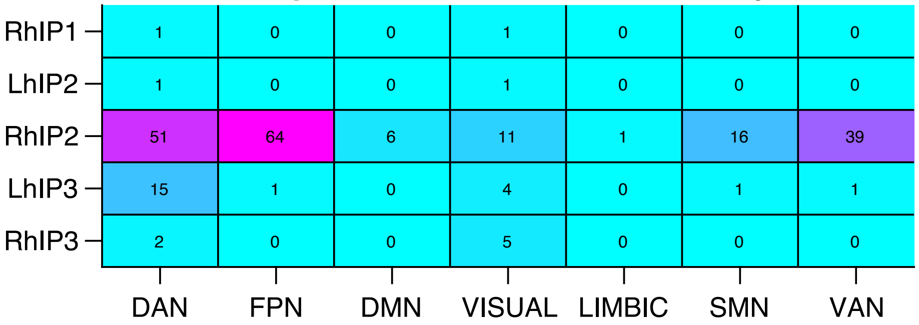

%Overlaps: the mental rotation system with the 7N

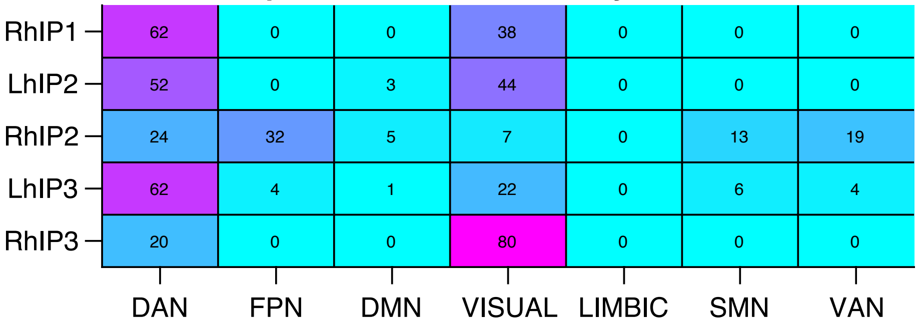

Supporting Information S6: Relative number of regions of the topic surface maps. Calculated as: (number of cortical regions of seed^{i} / total number of regions of all seed regions per hemisphere)*100.

	Seed Regions					
Systems	LhIP1	LhIP3	LhIP3	RhIP1	RhIP2	RhIP3
Working Memory	36.7	11.9	51.4	24.1	32.1	43.8
Numeric Cognition	29.7	29.4	40.9	24.1	31.6	44.4
Grasping	0.9	35.1	64.0	0	2.3	97.7
Mental Rotation	0	7.7	92.3	1.1	96.8	2.1
Recognition Memory	66.7	16.7	16.7	100	0	0
Language	47.4	15.8	36.8	100	0	0
Navigation	0	93.8	6.3	26.1	12.1	61.8
Motion/Shapes	7.7	92.3	0	0	0	100
Attention	35.5	41.9	22.6	34.1	12.1	53.8

Supporting Information S7: Topic surface maps and flatmaps of the functional systems associated with the seed-cortical pairs. Here, we decomposed the contribution of each seed in color gradings depicting either unique or overlapping (i.e., common) co-activations.

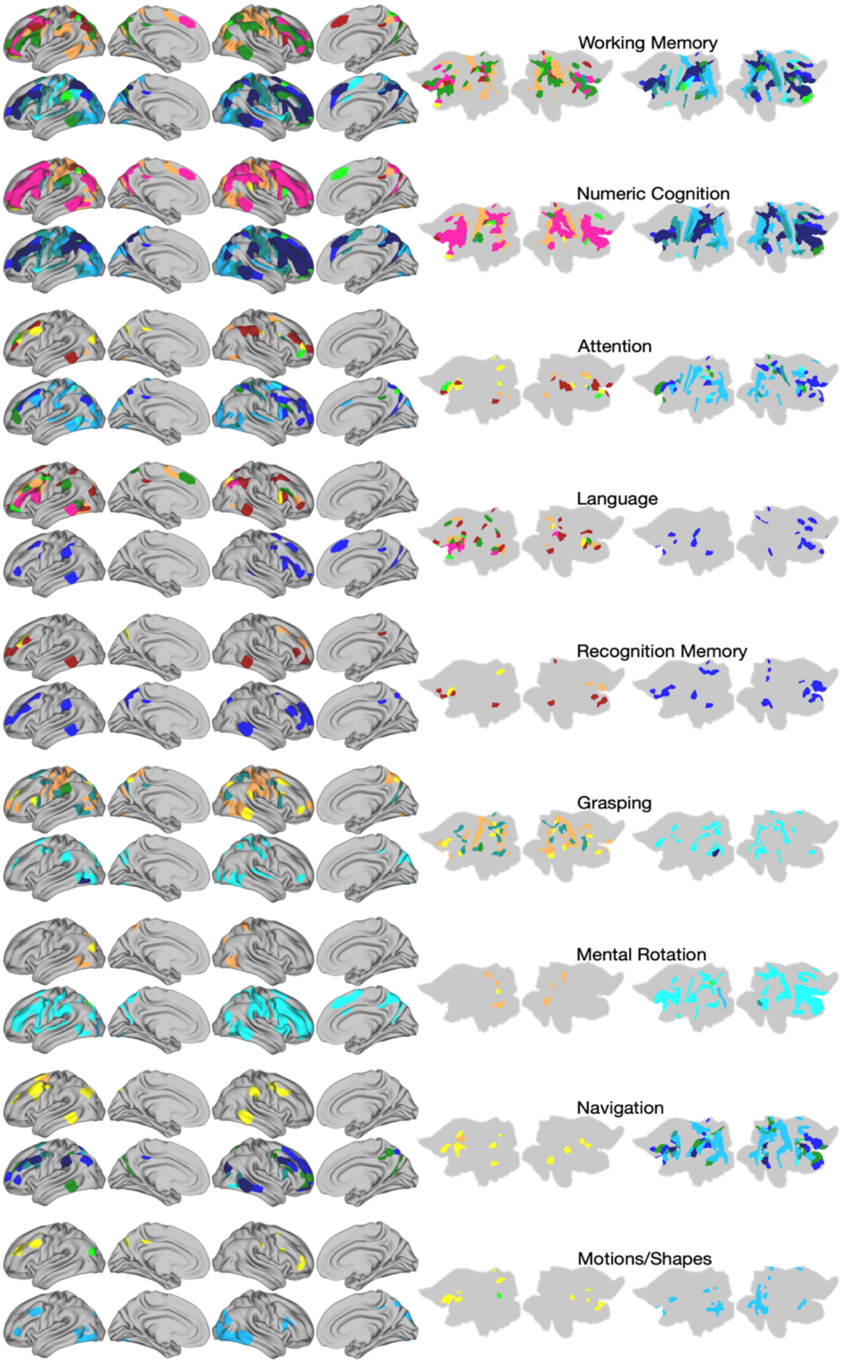
Working Memory
Numeric Cognition
Attention
Language
Recognition Memory
Grasping
Mental Rotation
Navigation
Motions/Shapes

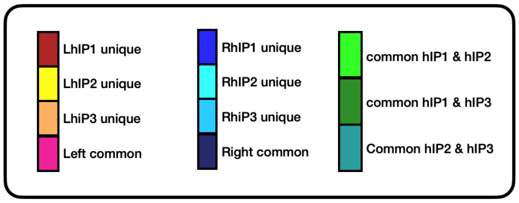
LhIP1 unique
LhIP2 unique
LhiP3 unique
Left common
RhIP1 unique
RhIP2 unique
RhiP3 unique
Right common
common hIP1 & hIP2
common hIP1 & hIP3
Common hIP2 & hIP3
